# Attenuation of Alzheimer’s brain pathology in 5XFAD mice by PTH_1-34_, a peptide of parathyroid hormone

**DOI:** 10.1186/s13195-023-01202-z

**Published:** 2023-03-14

**Authors:** Li Chen, Lei Xiong, Lingling Yao, Jinxiu Pan, Emily Arzola, Xiaojuan Zhu, Lin Mei, Wen-Cheng Xiong

**Affiliations:** 1 Department of Neurosciences, School of Medicine, Case Western Reserve University, 2210 Circle Dr, Cleveland, OH 44106 USA; 2 Institute of Cytology and Genetics, Northeast Normal University, Changchun, Jilin China; 3 Louis Stokes Cleveland Veterans Affairs Medical Center, Cleveland, OH USA

**Keywords:** Alzheimer’s disease, PTH, Aβ, Neuroinflammation, Astrocytes

## Abstract

**Background:**

Alzheimer’s disease (AD) and osteoporosis are two distinct diseases but often occur in the same patient. Their relationship remains poorly understood. Studies using Tg2576 AD animal model demonstrate bone deficits, which precede the brain phenotypes by several months, arguing for the independence of bone deficits on brain degeneration and raising a question if the bone deficits contribute to the AD development. To address this question, we investigated the effects of PTH_1-34_, a peptide of parathyroid hormone analog and a well-recognized effective anabolic therapy drug for patients with osteoporosis, on 5XFAD animal model.

**Methods:**

5XFAD mice, an early onset β-amyloid (Aβ)-based AD mouse model, were treated with PTH_1-34_ intermittently [once daily injection of hPTH_1–34_ (50 μg/Kg), 5 days/week, starting at 2-month old (MO) for 2–3 month]. Wild type mice (C57BL/6) were used as control. The bone phenotypes were examined by microCT and evaluated by measuring serum bone formation and resorption markers. The AD relevant brain pathology (e.g., Aβ and glial activation) and behaviors were assessed by a combination of immunohistochemical staining analysis, western blots, and behavior tests. Additionally, systemic and brain inflammation were evaluated by serum cytokine array, real-time PCR (qPCR), and RNAscope.

**Results:**

A reduced trabecular, but not cortical, bone mass, accompanied with a decrease in bone formation and an increase in bone resorption, was detected in 5XFAD mice at age of 5/6-month old (MO). Upon PTH_1-34_ treatments, not only these bone deficits but also Aβ-associated brain pathologies, including Aβ and Aβ deposition levels, dystrophic neurites, glial cell activation, and brain inflammatory cytokines, were all diminished; and the cognitive function was improved. Further studies suggest that PTH_1-34_ acts on not only osteoblasts in the bone but also astrocytes in the brain, suppressing astrocyte senescence and expression of inflammatory cytokines in 5XFAD mice.

**Conclusions:**

These results suggest that PTH_1-34_ may act as a senolytic-like drug, reducing systemic and brain inflammation and improving cognitive function, and implicate PTH_1-34_’s therapeutic potential for patients with not only osteoporosis but also AD.

**Supplementary Information:**

The online version contains supplementary material available at 10.1186/s13195-023-01202-z.

## Background

Alzheimer’s disease (AD) is the most common neurodegenerative disease that affects millions of people worldwide [1]. Clinically, it is characterized by progressive cognitive deterioration accompanied by multisystem dysfunction [2–5]. Pathologically, it features with increased β-amyloid (Aβ) plaques, hyperphosphorylated Tau^+^ neurofibrillary tangles, glial cell activation, brain inflammation, synaptic dysfunction, and neuron loss [1, 6]. Interestingly, in addition to brain pathologies, AD patients often have osteopenia or osteoporosis, a condition characterized by the loss of bone mass or bone mineral density (BMD) with deterioration of bone tissue micro-architectural, and a high incidence of hip fractures [7–10]. β-amyloid peptide and phosphorylated tau have been detected in tissues outside of the central nervous system, including osteoporotic bone tissue, and they are negatively correlated with BMD [11]. TgAPP_swe_^OCN^ mice, that selectively express Swedish mutant APP (APP_swe_) (a risk gene for early onset AD) [12] in osteoblast lineage cells, exhibit not only bone-loss but also AD-associated brain pathological and behavioral phenotypes [13, 14], suggesting a contribution of APP_swe_ induced bone deficits to the AD relevant brain pathology. However, the exact relationship between AD and osteoporosis remains elusive.

Parathyroid hormone (PTH) is a peptide secreted by the parathyroid glands that regulate serum calcium levels through its effects on bone, kidneys, and intestines [15, 16]. PTH analogs, such as PTH_1-34_, are considered to be the most effective and currently FDA-approved anabolic therapy for patients with osteoporosis [17]. PTH_1-34_’s effects on bone depend primarily on the mode of its treatment. Chronically sustained treatment of PTH_1-34_, acting as hyperparathyroidism, indirectly activates osteoclasts to exert a catabolic effect on bone mass, while intermittent PTH_1-34_ administration activates osteoblasts to a large extent, increasing bone formation and thus bone mass [17–19].

Although PTH_1-34_ is a therapeutic medicine for osteoporosis, studies emerged in recent years implicate PTH in neurodegenerative diseases [15, 20, 21]. PTH maintains bone balance and calcium levels in the body, and abnormal PTH levels may cause neuronal calcium imbalance, hypoperfusion, and disruption of neural signaling, showing neurodegenerative phenotypes [20, 22, 23]. PTH facilitates the conversion of vitamin D to its active form (1, 25-dihydroxyvitamin D), which may have a protective effect on neurons [24]. There are reports suggesting the association of hypoparathyroidism and hyperparathyroidism with the cognition decline [25, 26]. PTH deficiency is also associated with neuroinflammation, and the inflammatory cytokines, such as IL-1β, IL-1ra, and IL-6, are reported to impair PTH secretion [16, 27]. In light of these reports and our observations of earlier onset of bone deficits in AD animal models [13, 14, 28], we asked whether PTH_1-34_ could slow down AD pathology and/or progression.

Here, we used 5XFAD mice, a well-characterized AD animal model that expresses human mutant APP and presenilin genes under the control of Thy1-promoter, to test PTH_1-34_’s effect for the following reasons. First, 5XFAD mice exhibit early onset Aβ based brain pathologies and cognition deficits [1, 29]. Second, 5XFAD (at 5/6-MO) showed a reduced trabecular, but not cortical, bone mass, with a decrease in bone formation and an increase in bone resorption. Third, 5XFAD mouse serum samples had decreased PTH, exhibiting some features of hypoparathyroidism. Interestingly, upon intermittent treatments of PTH_1-34_, 5XFAD mice exhibited diminished not only bone deficits but also brain pathologies, including Aβ accumulation and deposition, glial cell activation, and brain inflammation, as well as improved learning and memory function. The PTH_1-34_ attenuation of AD brain pathology (e.g., Aβ accumulation and deposition and glial cell activation) was detectable in both female and male 5XFAD, but not wild type (WT), mice. However, PTH_1-34_’s improvement in learning and memory function was only observed in 5XFAD female mice at the age of ~ 5-MO. Further studies demonstrated that PTH_1-34_ was able to enter the brain tissue of 5XFAD mice and concentrated around astrocytes. In primary cultured astrocytes, PTH_1-34_ could induce phospho-CREB, a key signaling downstream of cAMP, suppresses expression of inflammatory cytokines or chemokines, and diminishes 5XFAD-induced astrocyte senescence. Taken together, these observations suggest that intermittent PTH_1-34_ treatments may act as a senolytic-like drug, reducing systemic and brain inflammation, improving cognitive function, and implicating the potential therapeutic benefits of PTH_1-34_ for not only osteoporosis but also AD.

## Materials and methods

### Animals

5XFAD [B6SJL-Tg (APPSwFlLon, PSEN1 * M146L * L286V) 6799Vas/Mmjax] transgenic mice were purchased from the Jackson Laboratory (Stock No: MMRRC_034840- JAX). 5XFAD mice express mutant human amyloid beta precursor protein (APP) and human presenilin 1 (PSEN1), with a total of five mutations including the Swedish [K670N/M671L], Florida [I716V], and London [V717I] in APP and M146L and L286V in PSEN1 under the control of Thy-1 promotor [1, 29]. 5XFAD mice were maintained as hemizygotes on a C57BL/6 background for experiment, and littermate controls (5XFAD-/-) or comparable aged C57BL/6J mice with same gender were used as WT control. The C57BL/6J mice (Stock No: 000664) were bred in our laboratory using mice purchased from The Jackson Laboratory. Tg2576 mice, which express human APP695 with the KM670/671NL mutations (APPswe) under the control of a hamster prion promoter [30], were purchased from Taconic (Hudson, NY, USA). Tg2576 mice were also backcrossed into C57BL/6 background, and WT (C57BL/6) control were used in parallel for each experiment. Female or male mice were subjected to experiments as indicated in the text. All mice were housed in groups of no more than 5 per cage with mice of the same genotype and gender under a 12 h light/dark cycle in a room with water and a standard rodent chow diet. All experiments with animals were approved by the Institutional Animal Care and Use Committee at Case Western Reserve University.

### Experimental design

Parathyroid hormone (1–34) (human) was purchased from TOCRIS Bioscience (Cat# 3011/1, Abingdon, United Kingdom). WT or 5XFAD mice (both male and female) were treated with PTH_1-34_ intermittently [once daily injection of hPTH_1–34_ (50 μg/Kg) or vehicle (0.9% sodium chloride) (as a control) by subcutaneous injection, starting at 2-MO, 5 days per week] as described previously [31]. Mice were then subjected to behavioral tests and sacrificed for examinations of their bone and brain phenotypes at the indicated ages.

### Micro-computed tomography (μCT)

Femurs of WT and 5XFAD female mouse with or without PTH_1-34_ treatment were detached and fixed overnight in 10% formalin and stored in PBS for further characterization by microCT (µCT). The excised femurs were placed vertically in a 12-mm-diameter scanning holder and scanned using the Scanco μCT40 (Scanco Medical AG). Settings parameters are adjusted as previously described: 12 μm resolution, 55 kVp, and 145 μA with an integration time of 200 ms [13, 32]. For the cortical analysis, the bone was scanned at the midshaft of the bone for a scan of 25 slices, and the threshold for cortical bone was set at 329. The 3D reconstruction was performed using all outlined slices and analyzed by μCT evaluation program (v.6.5–2; Scanco Medical). Data were obtained on bone volume (BV), total volume (TV), BV/TV, bone density, and cortical thickness for cortical bone. For the trabecular bone, the scan was started at the growth plate and consisted of 211 slices. One hundred slices were outlined from this point, on the inside of the cortical bone, enclosing only the trabecular bone and marrow. The threshold for trabecular bone was set at 245 and the 3D analysis performed on the 100 slices. BV, TV, BV/TV, trabecular bone number (Tb. N), trabecular bone thickness (Tb. Th), and trabecular bone space (Tb. Sp) were obtained for the trabecular bone.

### Behavior tests

Mice (female and male) at the age of ~ 5-MO were subjected to behavioral studies. The experimental procedures are described as previously [33, 34]. Mice were transferred to the behavior test room 2 h before any test to acclimatize to the environment. All behavioral instruments were cleaned with 70% ethanol before each trial. For Y-Maze test, mice were placed at one arm end of Y-maze and allowed to freely explore the three arms for 8 min. The total arm entries and the number of triads were recorded to quantify the percentage of spontaneous alternation. For novel object recognition (NOR), which tests mouse’s memory ability for a novel object, 20 min were recorded to analyze the time to explore the old object vs. the new object of each mouse. Mice were released into the chamber (60 × 60 × 20 cm, WxDxH) for 20 min to acclimatize on the first day, followed by the training and testing on the second day. Mice were free to explore two identical objects in the chamber for 20 min during the training phase. The test trials were administered after delays of 3-h post-training. During test trials, one of the objects was replaced with a new object and mice were free to explore for another 20 min, and the time spent exploring the new object and the old object was recorded. The discrimination index is quantified as the novelty object preference, which is the time spent exploring the new object minus the time exploring the old object over the total exploration time.

For Morris water maze (MWM), a circular water tank (diameter of 120 cm) fulfilled with water and a 10 cm platform were used. Non-toxic white powder paint was added to water to make the surface opaque to hide the escape platform. The maze was virtually divided into 4 quadrants, one of which contained the platform 1 cm below the water surface as an escape platform. Mice were trained for 5 days, four trials per day with 20 min intervals between trials and 60 s per trial to locate the hidden platform. Then mice were left on the platform for 15 s to help them remember the location. On the 6th day, the escape platform was removed and mice were placed in water at the point farthest from the platform and recorded for 60 s. The swimming track was recorded using a video tracking system, and the time spent in each platform quadrant and the number of platform crossings were quantified. All behavioral trials were recorded by use of an overhead camera and analyzed by Etho Vision software (Etho Vision, Noldus). Mice were assigned and data were quantified, in a double-blind method.

### Elisa assays for osteocalcin, PYD, human Aβ40 and Aβ42, and PTH

Submandibular blood collection was performed in anesthetized mice. A swift lancing motion is used to puncture the vessel. Blood sample (up to 200 µl) was collected with a pipette or other collection tube, allowed to stand at room temperature for 30 min and centrifuged for 15 min at 3000 rpm. Supernatant serum was aliquot and frozen at – 80 °C until use. Mouse serum levels of osteocalcin (a marker for bone formation), PYD (pyridinoline, a marker for bone resorption), and PTH were measured using mouse osteocalcin Elisa kit (QUIDEL Corporation), METRA Serum PYD EIA kit (QUIDEL Corporation, San Diego, CA, USA), and mouse PTH Elisa kit (MyBioSource), respectively, following the manufacturers’ instruction.

Brain homogenized lysates from the cortex and hippocampus were obtained for human Aβ40/42 Elisa assays, as previously described with modifications [14, 34]. In brief, the detached tissue was homogenized in modified ice-cold TBS buffer [50 mM Tris–Cl (pH 7.6), 150 mM NaCl, 1% NP-40] with Dounce homogenizers until no visible pieces were seen. The mixture was centrifuged at 12,000* g* for 20 min at 4 °C, and the supernatants were collected as soluble fractions for Aβ Elisa analysis. Followed by the manufacturer’s instruction, human Aβ40 and Aβ42 levels in the brain (200 μg total protein) homogenates were measured by an Aβ40 human ELISA kit (Invitrogen, Cat# KHB3481) and Aβ42 human ELISA kit (Invitrogen, Cat# KHB3441). All ODs measured after the reaction were converted to their concentrations using their corresponding standard curves.

For PTH Elisa assays, cortex and hippocampus from 6-MO WT and 5XFAD female mice were minced into small pieces and rinsed in ice-cold PBS to remove excess blood thoroughly. Tissue pieces were weighed and then homogenized in PBS [tissue weight (g): PBS volume (mL) = 1:9] with a glass homogenizer on ice. The homogenates were then centrifuged for 5 min at 5000* g* to get the supernatant. Mouse PTH levels were then measured by the PTH Elisa kit (MyBioSource, Cat# MBS2509255) according to instructions. The optical density (OD) was measured spectrophotometrically at a wavelength of 450 nm. The concentration of PTH were calculated by comparing the OD of the samples to the standard curve.

### Brain tissue processing and immunofluorescent staining

Coronal brain sections were obtained and subjected to immunofluorescent staining, as described previously [35]. In brief, mice were anesthetized by 3% isoflurane and transcardially perfused with phosphate buffer (PBS, 0.01 M, pH = 7.4) followed by 4% (w/v) paraformaldehyde (PFA), and the dissected brains were post-fixed with 4% PFA overnight at 4 °C. Brain tissues were sectioned into 40 μm-thick free-floating coronal sections for different purposes using a vibratome (Leica VT1000S). All brain slices were sequentially collected and stored at – 20 °C in cryoprotectant solution (FD Section Storage Solution) for further use.

For immunostaining, free-floating sections were washed in PBS (3–5 min, 3 times) and incubated in blocking buffer (0.02% Triton X-100, and 2% donkey serum in PBS) for 1 h, then incubated in primary antibody solution overnight at 4 °C. Sections were washed 3 times in PBS and incubated with corresponding conjugated secondary antibodies (1: 200 in blocking buffer) for 2 h at room temperature. DAPI was used for nucleus staining. Stained sections were imaged by a confocal microscope at room temperature. Fluorescent quantification was performed using ZEN software according to the manufacturer’s instructions (Carl Zeiss). The following primary antibodies were used: anti-ATG9A (ab108338, rabbit), anti-IBA1 (ab178846, rabbit), anti-IBA1 (ab5076, goat), and LPL (ab21356, mouse) from Abcam (Cambridge, MA, USA); anti-GFAP (#12389, rabbit) from Cell Signaling (Danvers, MA, USA); SLC16A1 (TA321556, rabbit) from OriGeine (Rockville, MD, USA); MAP2 (#556320, mouse) from BD Pharmingen (San Diego, CA, USA). Secondary antibodies were purchased from Jackson ImmunoResearch Laboratories (West Grove, PA, USA).

### Aβ deposition assay

Thioflavin S (Thio-S) staining was used for Aβ deposition assay as previously described with minimal modification [34]. Beginning with the lateral-most section in the ROI (region of interest), every sixth tissue section of six consecutive sections was used. Free-floating brain slices were washed in PBS (3–5 min, 3 times) and incubated in 0.1% Thio-S solution (dissolved in ddH_2_O and filtered) for 10 min at room temperature in the dark. The brain slices were then washed with a series of graded EtOH in the dark as follows: 95% EtOH for 2 min, 80% EtOH for 2 min, and 70% EtOH for 2 min. Finally, the brain slices were washed with PBS 3 times in the dark and transferred to slides for sealing with an antifade fluorescence mounting medium. All the brain slices were captured on a Laser confocal microscopy. The images were adjusted to the same threshold to increase signal-to-noise ratio. Plaques > 5.5 μm^2^ were quantified. Four sections per mouse were quantified. The layers or sub-regions of cortex and hippocampus in each image were outlined and analyzed with the National Institutes of Health ImageJ software.

### Western blot analysis

Total proteins from cortex, hippocampus, or cultured astrocytes were extracted using RIPA lysis buffer [50 mM Tris–HCl (pH7.5), 150 mM NaCl, 1 mM EDTA, 1% v/v NP-40, 0.1% SDS, 1% sodium deoxycholate supplemented with Protease Inhibitor Cocktail (Roche) and 1 mM PMSF] and analyzed by Western Blot. Lysates were cleared by centrifugation at 12,000* g* for 15 min at 4 °C to remove debris and to obtain homogenates. Homogenates samples were resolved by SDS-PAGE (8 ~ 15%) gels and then electroblotted onto nitrocellulose membranes (Cat# 1620112, Bio-Rad Laboratories). Membranes were blocked with 5% BSA (w/v) in TBS-Tween (50 mM Tris, 150 mM NaCl, pH7.6 and 0.1% v/v Tween-20) for 1 h and then probed with indicated primary antibodies overnight in 4 °C. Membranes were washed with TBST 4 times and incubated with HRP-conjugated secondary antibodies (1:5000 in TBST) at room temperature for 1 h. After washing with TBST 3 times, antibody reactivity was detected by the Enhanced Chemiluminescence (ECL) detection system (Amersham Biosciences). Band density of proteins was normalized in relation to loading control and analyzed using NIH ImageJ software. Primary antibodies used were as follows: IBA1 (ab178846, rabbit), p16 (ab51243, rabbit), p53 (ab131442, rabbit), and anti-beta Actin (ab8226, mouse) from Abcam (Cambridge, MA, USA); GFAP (12389, rabbit), p-CREB (Ser133) (9198S, rabbit), p-AKT(S473) (4060P, rabbit), AKT (9272S, rabbit) and GAPDH (97166S, mouse) from Cell Signaling (Danvers, MA, USA); PTH1R (PA5-77,689, rabbit) from Invitrogen (Carlsbad, CA, USA); p-ERK1/2 (sc-136521, mouse) and ERK1/2 (sc-514302, mouse) from Santa Cruz Biotechnology (Santa Cruz, CA, USA). Secondary antibodies were purchased from Jackson ImmunoResearch Laboratories (West Grove, PA, USA).

### Reverse transcription-quantitative polymerase chain reaction (qRT-PCR)

Total RNA was extracted from cortex, hippocampus, or cultured astrocytes using a TRizol reagent (Invitrogen, cat#15596–026, Carlsbad, CA, USA) as previously described [35]. Purified RNA (1–5 μg) was used for cDNA synthesis with the Revert Aid First Strand cDNA Synthesis Kit (Thermo Scientific, cat#K1621, Waltham, MA, USA). The cDNA products were subjected to subsequent quantitative PCR (qPCR) using the QuantiFast SYBR Green PCR Kit (QIAGEN, cat#204057, Hilden, Germany) with a qPCR System (StepOne Plus). Primers used were as follows: Il6, 5′- CTTGGGACTGATGCTGGTG-3′ and 5′- TTGGGAGTGGTATCCTCTGTGA-3′; Il10, 5′- CGGGAAGACAATAACTGCACCC-3′ and 5′- CGGTTAGCAGTATGTTGTCCAGC-3′; Il1β, 5′- TGGACCTTCCAGGATGAGGACA-3′ and 5′-GTTCATCTCGGAGCCTGTAGTG-3′;Tnfα, 5′- GGCGGTGCCTATGTCTCA-3′ and 5′-CCTCCACTTGGTGGTTTGT-3′; Tgfβ1, 5′-ACCGCAA CAACGCCATCT-3′ and 5′-GGGCACTGCTTCCCGAAT-3′; Ccl5, 5′- ACCACTCCCTGCTGC TTT-3′ and 5′- ACACTTGGCGGTTCCTTC-3′; Gm-csf, 5′- AACCTCCTGGATGACATGCCTG-3′ and 5′- AAATTGCCCCGTAGACCCTGCT-3′; Tnfsf11 (Rank), 5′-GGACAACGGAATCAGATGTGGTC-3′ and 5′-CCACAGAGATGAAGAGGAGCAG-3′; Rankl, 5′-ATCCCATCGGGTTCCCATAA-3′ and 5′-TCCGTTGCTTAACGTCATGTTAG-3′; Tnfrsf11b (Opg), 5′-GGCCTGATGTATGCCCTCAA-3′ and 5′-GTGCAGGAACCTCATGGTCTTC-3′; Cdkn2a (p16^Ink4a^), 5′-TGTTGAGGCTAGAGAGGATCTTG-3′ and 5′- CGAATCTGCACCGTAGTTGAGC′-3′; Trp53, 5′-TGAAGCACCGCTTCCCGAAGAG-3′ and 5′-AGAAGACGACTGGGGCAGCTAT-3′. Each sample was repeated at least 3 times, and the mRNA level was normalized to GAPDH using the 2^−△△Ct^ method.

### Mouse Serum Cytokine array

Serum samples from 5XFAD, Tg2576, and their WT control female mice (6 ~ MO) were collected and used for serum cytokine detection. Cytokines were measured with Mouse Cytokine Array Panel A (R&D Systems, cat#ARY006, Minneapolis, MN, USA). Briefly, the mixture of serum and the biotinylated detection antibodies cocktail is incubated with a Mouse Cytokine Array membrane. Any cytokine/detection antibody complex present was bound on the membrane by its cognate immobilized capture antibody. After washing off the unbound protein, streptavidin–horseradish peroxidase and chemiluminescent detection reagents were added to generate light signals. The light produced at each spot is proportional to the amount of cytokine binding.

### Biotin-PTH_1-34_ diffusion experiment

Biotin-labeled PTH_1-34_ (ANASPEC, cat# AS-20690, Fremont, CA, USA) was used to detect PTH diffusion and function. 2.5 ~ MO old WT and 5XFAD female mice were anesthetized and given a total of 100 μg of Biotin-PTH_1-34_ or vehicle (Phosphate Buffer) by tail-intravenous injection. After 30 min, mice were perfused with 4% PFA (pH 7.4), and the brain slices were obtained as described above. Immunofluorescent staining was used to label the biotin signal using a biotin antibody. All the brain slices were captured on laser confocal microscopy.

### Astrocyte culture and in vitro PTH_1-34_ treatment

Astrocyte culture was performed according to the standard protocol with some modifications as described in previous literature [36–38]. In brief, the cerebral cortex and hippocampus of postnatal day 3 (P3) female pups were dissected under a stereomicroscope and placed in ice-cold calcium- and magnesium-free HBSS (Hanks Balanced Salt Solution). The tissues were digested with 2.5% trypsin–EDTA, 3 ml for each mouse, at 37 °C for 20 min with shaking slightly every 5 min. The digested tissue was blown into single-cell suspension, filtered through a 70-mm filter mesh, pelleted at 900 × *g* for 5 min, and resuspended in growth medium (DMEM plus 10% FBS), which was incubated at 37 °C with 5% CO_2_. Three cortical and hippocampal tissues per 100 mm culture dish were necessary to achieve a proper astrocyte density. The culture medium was changed after 24 h and every 3 days following the initial change to remove non-astrocyte cells. Confluent cells were passed after 7 days using 0.05% trypsin–EDTA and could be used for experiments after another 3–6 days of culture. Cultured astrocytes were treated with 100 ng/ml of PTH_1-34_, and samples were collected at indicated times to detect changes in proteins, genes, and cells.

### RNA scope

The RNA scope was performed in mouse brain using the RNAscope® Multiplex Fluorescent Detection Kit (Cat#PN323110, Noble Park North, Australia). The probes and hybrid oven were purchased from the ACDbio company, and all the procedures were performed according to the manufacturer’s protocol and as described previously [38]. Briefly, 6 ~ MO WT and 5XFAD female mice were deeply anesthetized and perfused with 4% PFA. The dissected brains were post-fixed with 4% PFA overnight and dehydrated with 30% sucrose in PBS. Then, the brains were cryo-sectioned into 12 μm sections using a freezing microtome (Leica, Buffalo Grove, IL, USA). Every sixth tissue section of six consecutive sections in ROI was used for staining and followed the RNAscope® Multiplex Fluorescent Reagent Kit v2 User Manual processing. Finally, sections were imaged with Zeiss LSM 800 system.

### Senescence associated β-Gal staining

SA-β-gal staining of cultured astrocytes was performed as previously reported [14]. SA-β-gal staining was performed using an SA-β-gal staining kit (Cell Signaling, cat#9860, Danvers, MA, USA) according to the manufacturer’s instructions. All stained cell slides were captured on a BZX slider scanner. The SA-β-gal intensity was quantified by the NIH ImageJ software.

### Statistical analyses

All data were presented as mean ± SD. For in vivo studies, three to eight mice per group per assay were used, and the gender of the mice was indicated in the experiment. For behavior tests, 6 ~ 8 mice per group were used, and the tests were conducted separately for different gender. For in vitro cellular biological and biochemical studies, each experiment was repeated at least three times. The numbers of biological replicates are described in the figure legends. All immunofluorescence staining and immunoblotting data were quantified by the NIH ImageJ software. Statistical analyses were performed using Prism 7.0 (GraphPad Software). For two independent data comparisons, unpaired Student’s *t*-test was used to determine statistical significance. For multiple comparisons of three or more sets of samples, one-way ANOVA or two-way ANOVA test were used. Differences were considered significant when *P* < 0.05 (*P < 0.05, **P < 0.01, ***P < 0.001).

## Results

### Trabecular bone loss in 5XFAD mice, which was attenuated by PTH_1-34_ treatments

**Fig. 1:**
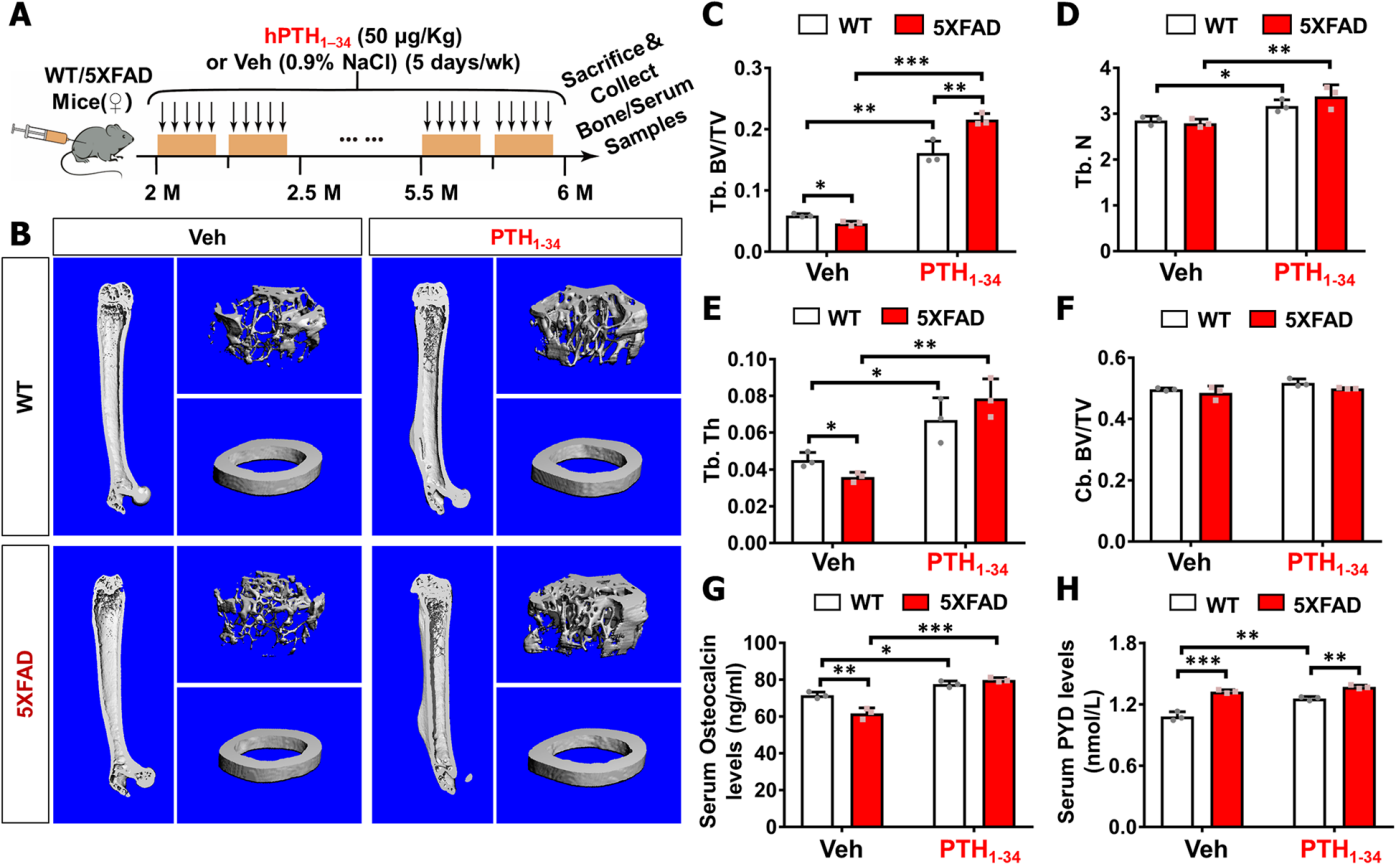
Trabecular bone loss in 5XFAD mice was diminished by PTH_1-34_ treatments. **A** Schematic of hPTH_1-34_ or vehicle (0.9% sodium chloride) intermittent treatment and tissue samples collection in WT/5XFAD female mice. Female WT/5XFAD mice were administered a once-daily injection of hPTH_1–34_, or veh (0.9% NaCl) via subcutaneous injection, starting at 2 ~ MO old, 5 days per week. Mice were sacrificed at 6 ~ MO old to detect bone phenotypes. **B**–**F** μCT analysis of femurs from 6 ~ MO WT and 5XFAD female mice with PTH_1-34_ or Veh treatment. Representative images are shown in **B** and quantification analyses of trabecular bone volume over total volume (Tb. BV/TV), trabecular bone number (Tb. N), trabecular bone thickness (Tb. Th), and cortical bone volume over total volume (Cb. BV/TV) by the direct model of μCT analysis are presented in **B**–**F**. **G** Serum osteocalcin levels analyses, measured by ELISA assays, in 6 ~ MO female mice. **H** Serum PYD levels analyses, measured by ELISA assays, in 6 ~ MO female mice. Three different female mice from each group were examined. The data were presented as mean ± SD, *P < 0.05, **P < 0.01, ***P < 0.001, two-way ANOVA with two-stage step-up method of Benjamini, Krieger, and Yekutieli multiple comparisons test was used

To further address whether AD-relevant mouse models show any skeletal bone deficit and to investigate whether PTH_1-34_ treatments affect AD-associated bone deficits, we used 5XFAD mouse, a well-characterized AD animal model that expresses mutant human APP and presenilin genes under the control of Thy1-promoter, and exhibit early onset Aβ based brain pathologies (~ 2 MO) and cognition deficits (~ 4 MO) [1, 29, 39]. C57BL/6J mice were used as WT controls. We first examined bone phenotypes by microCT (μCT) analyses in femurs of control/WT and 5XFAD female mice, in light of reports of earlier onset and severer phenotypes in female than male 5XFAD mice [29, 40]. The control/WT and 5XFAD mice were treated with Vehicle (0.9% NaCl) or PTH_1-34_ intermittently (once per day and five days per week), starting at age of 2-MO and sacrificed at ~ 6-MO, as illustrated in Fig. 1A. Notice that in the absence of PTH_1-34,_ μCT examinations showed reductions in trabecular bone volumes (Tb. BV/TV) and trabecular bone thickness (Tb. Th), without changes in their trabecular bone numbers (Tb. N) and cortical bone volumes (Cb. BV/TV), in 5XFAD mice (at age of 6-MO), as compared with those of litter mate control/WT mice (Fig. 1B–F), suggesting a bone deficit in 5XFAD mice. In line with this view were observations that serum levels of osteocalcin (a marker for bone formation) were decreased, but PYD (pyridinoline) (a maker for bone resorption) levels were increased, in 5XFAD mice (Fig. 1G–H). These results thus implicate both reduced bone formation and elevated bone resorption to underlie the trabecular bone-loss in 5XFAD mice.

Interestingly, upon PTH_1-34_ treatments, more trabecular bone mass was induced in 5XFAD mice than that in WT mice (Fig. 1B–C), suggesting an enhanced PTH_1-34_ response in 5XFAD mice. In line with this view were the observations that PTH_1-34_ increased serum osteocalcin levels more in 5XFAD mice than that in control mice (Fig. 1G), but had little to no effect on serum PYD levels in 5XFAD mice, as compared to that of WT controls (Fig. 1H). These results thus suggest that the PTH_1-34_-induced trabecular bone mass in 5XFAD mice is likely due in large part to the elevated bone formation. The two-way ANOVA analysis showed that PTH_1-34_ treatment has a significant effect on various measures of bone phenotypes (*P* < 0.05) and that genotype has a significant effect on Tb. BV/TV, serum osteocalcin levels, and serum PYD levels (*P* < 0.05) (Supplemental file 1 Table 1). Significant interactions between PTH_1-34_ treatment x genotype have been observed in Tb. BV/TV (*F* = 27.03, *p* = 0.0008), serum osteocalcin levels (*F* = 26.59, *p* = 0.0009), and serum PYD levels (*F* = 17.01, *p* = 0.0033) (Supplemental file 1 Table 1). These results suggest an enhanced bone metabolism in response to PTH_1-34_ in 5XFAD mice.

To understand how PTH_1-34_ induced anabolic response is enhanced in 5XFAD mice, we measured mouse PTH levels in control and 5XFAD female mouse serum samples and brain (cortex and hippocampus) homogenates (at age of 6 ~ MO). Interestingly, ELISA showed lower levels of PTH in 5XFAD serum and hippocampal samples than those of controls but comparable levels of PTH in 5XFAD cortex to that of controls (Fig. S1A-B). These results suggest that PTH is detectable in the brain, but it is reduced in 5XFAD serum and hippocampus. Such a PTH-deficiency may underlie the enhanced response to the injected hPTH_1-34_.

### PTH_1-34_ attenuation of cognition decline and memory-loss in 5XFAD mice

**Fig. 2:**
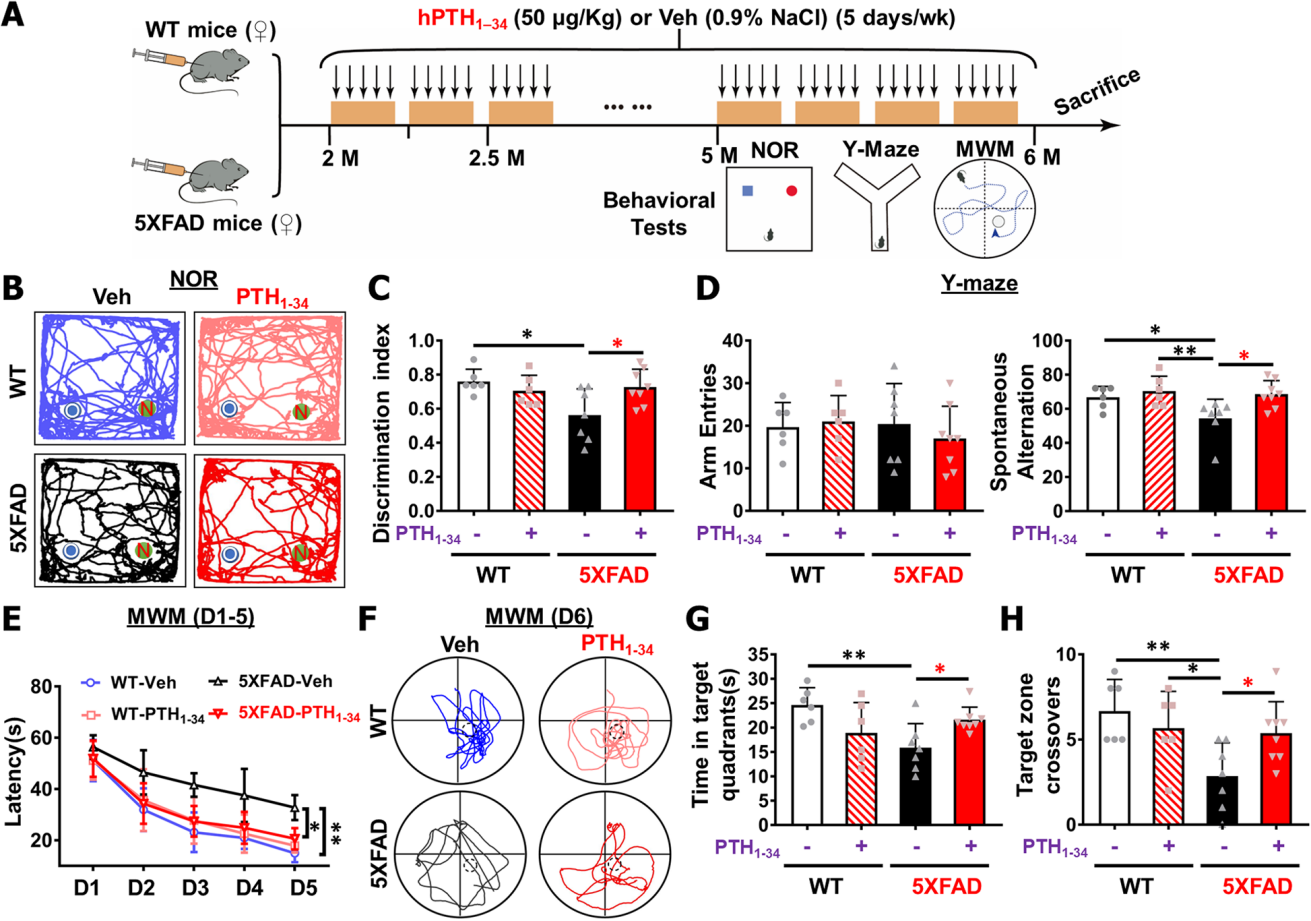
PTH_1-34_ attenuation of cognition decline and memory loss in 5XFAD female mice. **A** Illustration of PTH_1-34_ or vehicle intermittent treatment in WT/5XFAD female mice and behavioral testing schedule. All mice were tested for behavior from the age of 5 ~ MO old and treated with PTH_1-34_/Veh continuously during the testing procedure. The results shown in this figure were for female mice. **B**, **C** Novel object recognition (NOR): Representative tracing images (**B**), and the quantification of discrimination index ([*n*ovel object explore *time* − *old* object explore *time*]/[*total explore time*]) of NOR (**C**) were shown. **D** Y maze: Quantifications of the total arm entries and spontaneous alternation. **E**–**H** Morris water maze (MWM): The latencies to reach the hidden platform during the training period (**E**), the representative tracing images (**F**), quantification of time spent in the target quadrant (**G**), and target zone crossovers (**H**) on the testing day were showed. All quantification data were presented as mean ± SD (*n* = 6–8 female mice per group). *P < 0.05, **P < 0.01, two-way ANOVA with two-stage step-up method of Benjamini, Krieger, and Yekutieli multiple comparisons test was used

AD is a progressive neurodegenerative disease commonly associated with memory deficits and cognitive decline [41]. We thus asked whether PTH_1-34_ treatments could improve cognitive function in 5XFAD mice. WT or 5XFAD female mice were administered with hPTH_1–34_ or Veh intermittently as illustrated in Fig. 2A, and behavioral experiments began when the mice were ~ 5-MO. Novel object recognition (NOR) and Morris water maze (MWM) tests were used to access mouse cognitive function or learning and memory [42], and Y-maze was used to test mouse working memory [43]. While the cognitive function was substantially reduced in 5XFAD female mice compared to WT mice of the same age, these declines were largely brought down by PTH_1-34_ (Fig. 2B–H). Upon treatments with PTH_1-34_, 5XFAD female mice performed significantly better during these tests, as compared with those of 5XFAD control mice, exhibiting improved recognition of the novel object (Fig. 2B–C), faster learning of the hidden platform during MWM tests (Fig. 2E), and better spatial memory of the platform location (Fig. 2F–H). Additionally, these PTH_1-34_ treated 5XFAD female mice showed an increase in spontaneous alternation, but comparable arm entries to those of Veh-treated 5XFAD female mice, during Y-maze tests (Fig. 2D), suggesting an improvement in spatial working memory. The two-way ANOVA analysis showed the effect of genotype (5XFAD) in the Y maze (spontaneous alternation: *F* = 4.283, *P* = 0.0499) and MWM test (target zone crossovers: *F* = 7.363, *P* = 0.0124; time in target quadrant: *F* = 3.095, *P* = 0.0918), while the significant effect of PTH_1-34_ treatment only detected in the Y maze (spontaneous alternation: *F* = 6.832, *P* = 0.0155). There were interactive effects between PTH_1-34_ treatment x genotype on NOR (discrimination index: *F* = 6.379, *P* = 0.0189) and MWM test (target zone crossovers: *F* = 5.417, *P* = 0.0291; time in target quadrant: *F* = 11.15, *P* = 0.0028) (Supplemental file 1 Table 2). The PTH_1-34_ improvement of the cognitive function in 5XFAD mice appeared to be more pronounced in females than males (Fig. 2 and Fig. S2). Little to no improvement in cognitive function in novel object recognition (Fig. S2A-B) nor spatial learning and memory in MWM (Fig. S2D-G) was detected by PTH_1-34_ treatments in 5XFAD male mice, although PTH_1-34_ had a positive effect on the spontaneous alternation during Y maze tests (Fig. S2C). In aggregates, these intriguing results suggest that PTH_1-34_ improves cognitive function in 5XFAD mice, which is currently manifested primarily in female mice.

### PTH_1-34_ reduction of soluble Aβ level and in-soluble Aβ deposition in 5XFAD brain

**Fig. 3:**
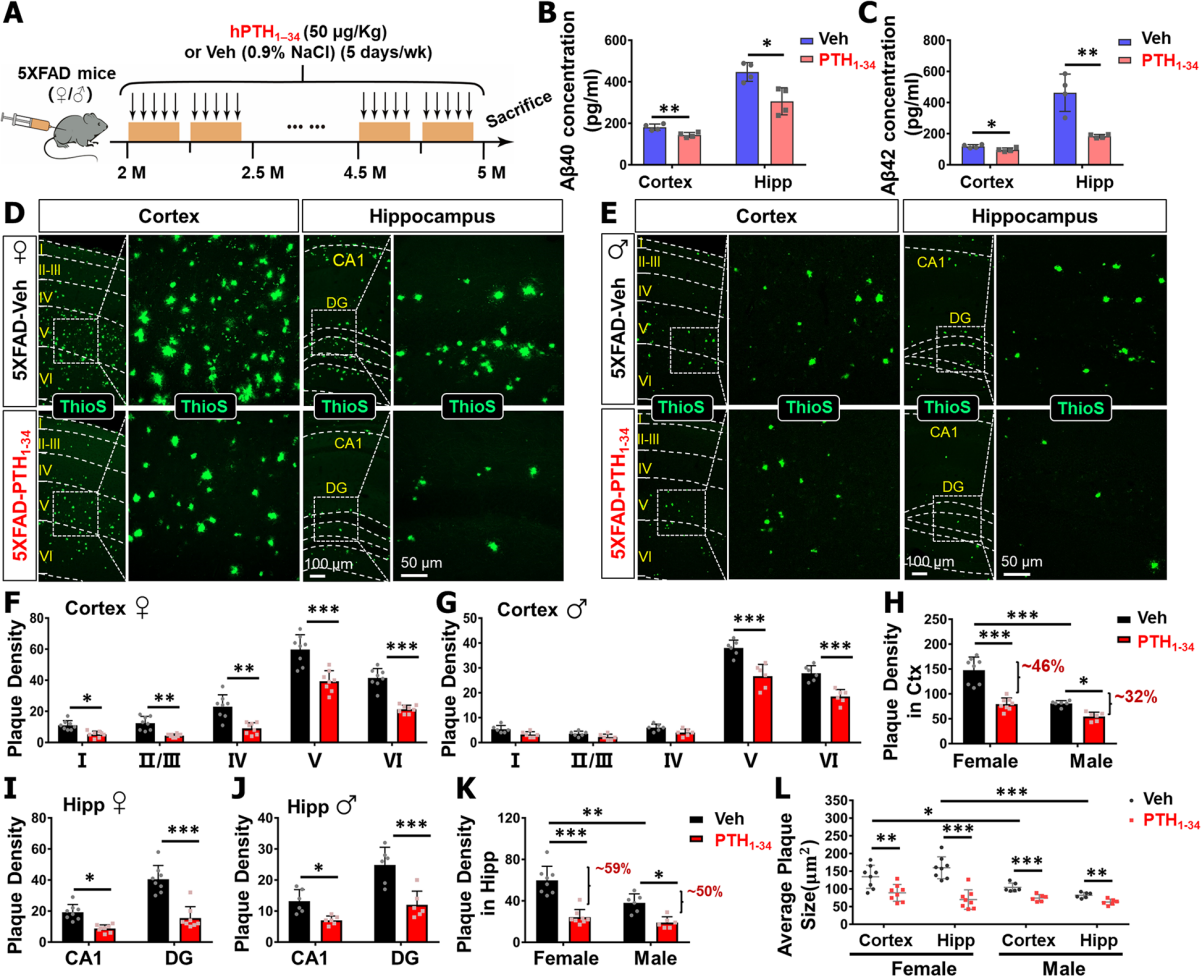
PTH_1-34_ reduction of soluble Aβ level and in-soluble Aβ deposition in 5XFAD brain. **A** Illustration of PTH_1-34_ intermittent treatment in 5XFAD mice. Mice were sacrificed at 5 ~ MO old to detect Aβ level and accumulation. **B**, **C** ELISA analyses of human Aβ40(B) and Aβ42(C) levels in the soluble fraction of brain homogenates including cortex and hippocampus (200 μg total protein) from female 5XFAD mice with PTH_1-34_ or Veh treatment (*n* = 4 mice per group). **D**, **E** Representative images of ThioS staining for Aβ plaque depositions analysis in the cortex and hippocampus of 5XFAD-Veh (control) and 5XFAD-PTH_1-34_ mice. Representative images of female mice were shown in **D** and representative images of male mice were shown in **E**. **F**, **G** Quantification of plaque density (the amount of plaque deposition in each sub-region) in the cortex of 5XFAD female and male mice. **H** Quantification of the total plaque density in the cortex of female and male 5XFAD mice. **I**, **J** Quantification of plaque density in subregions of hippocampus in 5XFAD female and male mice. **K** Quantification of the total plaque density in the hippocampus of female and male 5XFAD mice. **L** Quantification of average plaque size in 5XFAD female and male mice. *n* = 8 per group for female mice and *n* = 6 per group for male mice in **F**–**L**. Scale bars as indicated in each panel. All data were presented as mean ± SD. *P < 0.05, **P < 0.01, ***P < 0.001, two-way ANOVA with Sidak’s multiple comparisons test was used

It is known that the β-amyloid accumulation and deposition in 5XFAD mice is a crucial pathology for AD development [29]. To investigate the effect of PTH_1-34_ treatments on AD-relevant brain pathology, 5XFAD mice (both male and female) were administered with hPTH_1–34_ or vehicle (Veh) as illustrated in Fig. 3A. We first measured both human Aβ_40_ and Aβ_42_ levels in soluble fraction from brain homogenates (cortex and hippocampus) of 5XFAD female mice in response to PTH_1-34_ treatments. ELISA analyses showed significant reductions in human Aβ_40_ and Aβ_42_ levels in homogenates of both cortex and hippocampus from 5XFAD mice treated with PTH_1-34_, as compared with those of Veh treatments (Fig. 3B–C), suggesting PTH_1-34_’s inhibitory effect on Aβ accumulation. To determine whether the insoluble human Aβ and mouse Aβ levels are altered in 5XFAD mice by PTH_1-34_ treatments, we then evaluated Aβ plaques or deposition in both groups of brain sections by Thio-S staining. In line with the view of PTH_1-34_ inhibition of Aβ accumulation, PTH_1-34_ treatments decreased Thio-S positive ( +) Aβ plaques in cerebral cortex and hippocampus of 5XFAD female mice; both plaque density in each layer of cortex or sub-regions of hippocampus and plaque size showed significant reductions, as compared with those of Veh-treated 5XFAD female mice (Fig. 3D, F, I, L). Notice that this PTH_1-34_ effect was detected in not only female but also in male 5XFAD mice (Fig. 3D–E). Plaque density (mainly in the fifth and sixth layers of the cortex and in the DG region of the hippocampus) (Fig. 3G, J) and plaque size (Fig. 3L) were all decreased in PTH_1-34_-treated 5XFAD male mice, as compared with Veh-treated male mice, indicating PTH_1-34_’s gender independence in this pathology. Remarkably, in the absence of the PTH_1-34_, the levels of amyloid deposition (both plaque density and plaque size) in the cortex and hippocampus of female 5XFAD mice were significantly higher than those in male 5XFAD mice (Fig. 3H, K, L). Interestingly, PTH_1-34_ reduction of the levels of amyloid deposition appeared to be more dramatically in female than those of male 5XFAD mice, with ~ 46% and ~ 59% reductions in plaque density in female 5XFAD cortex and hippocampus, respectively, while ~ 32% and ~ 50% reductions in male 5XFAD cortex and hippocampus, respectively, and ~ 34% and ~ 56% reductions in plaque size in female 5XFAD cortex and hippocampus, respectively, while ~ 28% and ~ 23% reductions in male 5XFAD cortex and hippocampus respectively (Fig. 3H, K, L). Significant effects of gender and PTH_1-34_ treatment were showed in both plaque density and plaque size (*P* < 0.05), no matter in the cortex or hippocampus (Supplemental file 1 Table 3). Significant interactions between PTH_1-34_ treatment x gender were observed in both plaque density (cortex: *F* = 11.17, *P* = 0.0027; hippocampus: *F* = 5.021, *P* = 0.0346) and plaque size (only hippocampus: *F* = 16.2, *P* = 0.0005) (Supplemental file 1 Table 3), indicating a sex difference in response to PTH_1-34_ treatment. Together, these results suggest that intermittently PTH_1-34_ treatments attenuate Aβ pathology in 5XFAD brain, and this effect is more obvious in female mice.

### PTH_1-34_ reduction of plague-associated dystrophic neurites, but not plaque-associated microglial cells in 5XFAD mice

To understand how PTH_1-34_ suppresses Aβ accumulation and deposition in the 5XFAD brain, we further examined Aβ plaque-associated pathology in 5XFAD female mice (~ 6 MO) treated with PTH_1-34_ or vehicle. Aβ plaques are surrounded by dystrophic neurites, a feature of neurodegenerative pathology, and activated glial cells, including IBA1^+^ microglia and GFAP^+^ astrocytes [44]. Co-immunostaining analysis of ThioS with ATG9A, a pre-autophagosome protein that accumulates in the dystrophic neurites and is commonly used as a marker for dystrophic neurites [44], showed smaller and less dense ATG9A^+^ dystrophic neurites in PTH_1-34_ treated 5XFAD brain (both cortex and hippocampus) than those of Veh-treated 5XFAD mice (Fig. S3A-B), indicating a reduction of dystrophic neurite formation by PTH_1-34_, in line with the view of PTH_1-34_ decrease of Aβ plaques.

We then performed a co-immunostaining analysis of ThioS with IBA1, a marker for microglial cells. Although the overall IBA1 fluorescence intensity was lower in PTH_1-34_ treated 5XFAD cortex and hippocampus than that of Veh controls (Fig. S4A-B), the ThioS^+^ Aβ plaque associated IBA1 fluorescence was unchanged by PTH_1-34_ treatments (Fig. S4D-E). Additionally, the IBA1 + cell density and fluorescence intensity in regions without Aβ plaques were also comparable in PTH_1-34_ treated 5XFAD mice to those of Veh treatments (Fig. S4C). These results suggest that the overall reduction in IBA1 fluorescence intensity may be due to the reduced number of Aβ plaques.

Notice that the Aβ plaque-associated microglial cells are also called DAM (Degeneration Associated Microglia), because of their unique distribution pattern and expression of molecular features (e.g., higher expressions of genes such as LPL, but lower levels of TMEM119 and CX3CR1, than those of Aβ-un-associated microglia or resting microglia) [33, 45]. As DAMs are implicated in promoting Aβ clearance [33, 46], we further examined whether DAMs are affected by PTH_1-34_ treatments. Co-immunostaining analysis of IBA1 and LPL (a marker for DAM) with ThioS showed little to no difference of Aβ associated LPL^+^IBA1^+^ microglial cells in the brain between PTH_1-34_ and Veh-treated 5XFAD mice (Fig. S4F-G), suggesting a little effect on DAM formation by PTH_1-34_. These results demonstrate decreased amyloid plaques accompanied by decreased neural toxicity in PTH_1-34_ treated 5XFAD mice, suggesting PTH_1-34_’s function in preventing both Aβ accumulation and dystrophic neurites formation.

### PTH_1-34_ diminishment of GFAP^+^ reactive astrocytes and brain inflammation in 5XFAD mice

**Fig. 4:**
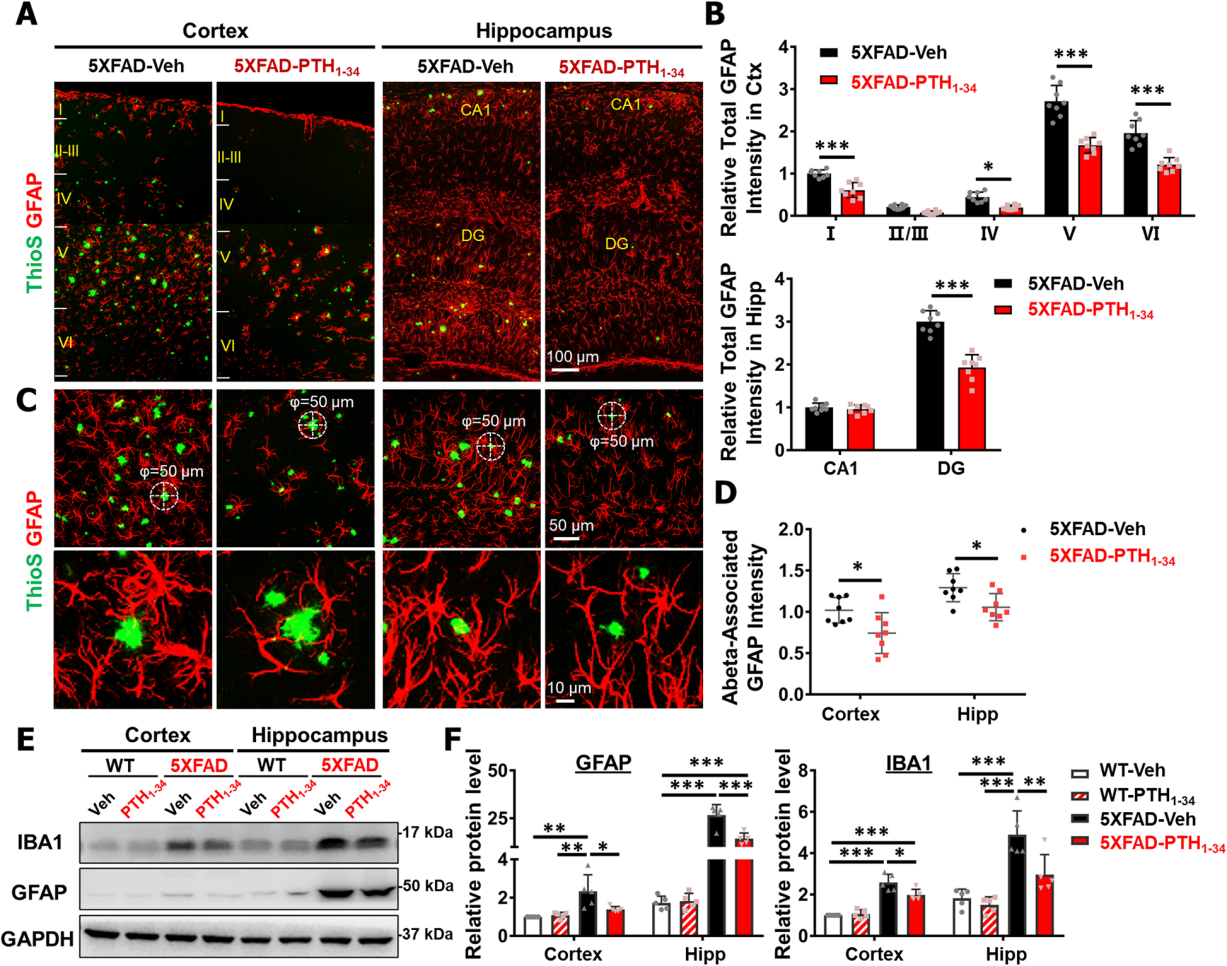
PTH_1-34_ diminishment of GFAP + reactive astrocytes. **A** Representative overall images of co-immunostaining with ThioS (green) and GFAP (red) of cortex and hippocampus from 6 ~ MO 5XFAD-Veh and 5XFAD-PTH_1-34_ female mice. **B** Quantification of relative GFAP fluorescence intensity in each layer of cortex and subregional of hippocampus. **C** Representative images and high-magnification images in Aβ deposition regions of co-immunostaining with ThioS (green) and GFAP (red) of cortex and hippocampus from 6 ~ MO 5XFAD-Veh and 5XFAD-PTH_1-34_ female mice. **D** Quantification of Abeta-associated GFAP fluorescence intensity. The Abeta-associated GFAP fluorescence intensity was defined by the intensity of GFAP-positive astrocytes in a plaque-centered circle within 50 μm in diameter (marked by dashed white circles). *n* = 8 mice per group. **E** Representative Western blots using antibodies against IBA1 and GFAP in homogenates of cortex and hippocampus from 6 ~ MO WT and 5XFAD female mice with PTH_1-34_ or Veh treatment. GAPDH was used as a loading control. **F** Quantification of relative protein level in **E** (*n* = 5 mice per group). All quantification data were presented as mean ± SD. Scale bars were indicated in each panel. *P < 0.05, **P < 0.01, ***P < 0.001, two-way ANOVA with Sidak’s multiple comparisons test was used

To further identify the effect of PTH_1-34_ on Aβ plaque-associated pathology, we examined GFAP^+^ astrocytes in female 5XFAD brains with PTH_1-34_ or Veh treatments (~ 6 MO). As shown in Fig. 4A–B, co-immunostaining analysis of GFAP (a marker for reactive astrocytes in cortex) with ThioS showed a similar overall response as those of IBA1^+^ microglial cells to PTH_1-34_ treatments. The increase in GFAP + reactive astrocytes was found in areas of Aβ deposition in 5XFAD-Veh mice, including each layer of the cortex, as well as the DG region of the hippocampus. The GFAP^+^ fluorescence intensity in layers of cortex (except layer II/III) and DG region of hippocampus were lower in PTH_1-34_ treated 5XFAD mice than that of Veh controls (Fig. 4A–B), in correlation with the reduced Aβ plaques by PTH_1-34_ treatments. However, in contrast to unchanged Aβ-plaque associated IBA1/LPL fluorescence (Fig. S4D-G), the plaque-associated GFAP fluorescence appeared to have diminished by PTH_1-34_ treatments (Fig. 4C–D). There were significant numbers of ThioS + plaques with little or no GFAP^+^ astrocytes surrounding the brain (cortex and hippocampus) of PTH_1-34_ treated 5XFAD mice (Fig. 4C). As the overall fluorescence intensity of IBA1 and GFAP decreased in PTH_1-34_ treated 5XFAD brain, we further tested this view by Western blot analysis. Certainly, lower IBA1 and GFAP protein levels were detected in PTH_1-34_ treated 5XFAD brain homogenates (cortex and hippocampus) (Fig. 4E–F), supporting the view for an overall reduction in glial reactivation by PTH_1-34_. Notice that this PTH_1-34_ inhibition of glial cell activation was detectable in both female and male 5XFAD mice (Figs. 4, S5); but PTH_1-34_ had little effect in these glial cells in wild-type (WT) mice (Fig. S6). These results thus suggest PTH_1-34_’s inhibitory effect on both astrocyte and microglial activation in 5XFAD, but not WT, mice in a sex-independent manner.

**Fig. 5:**
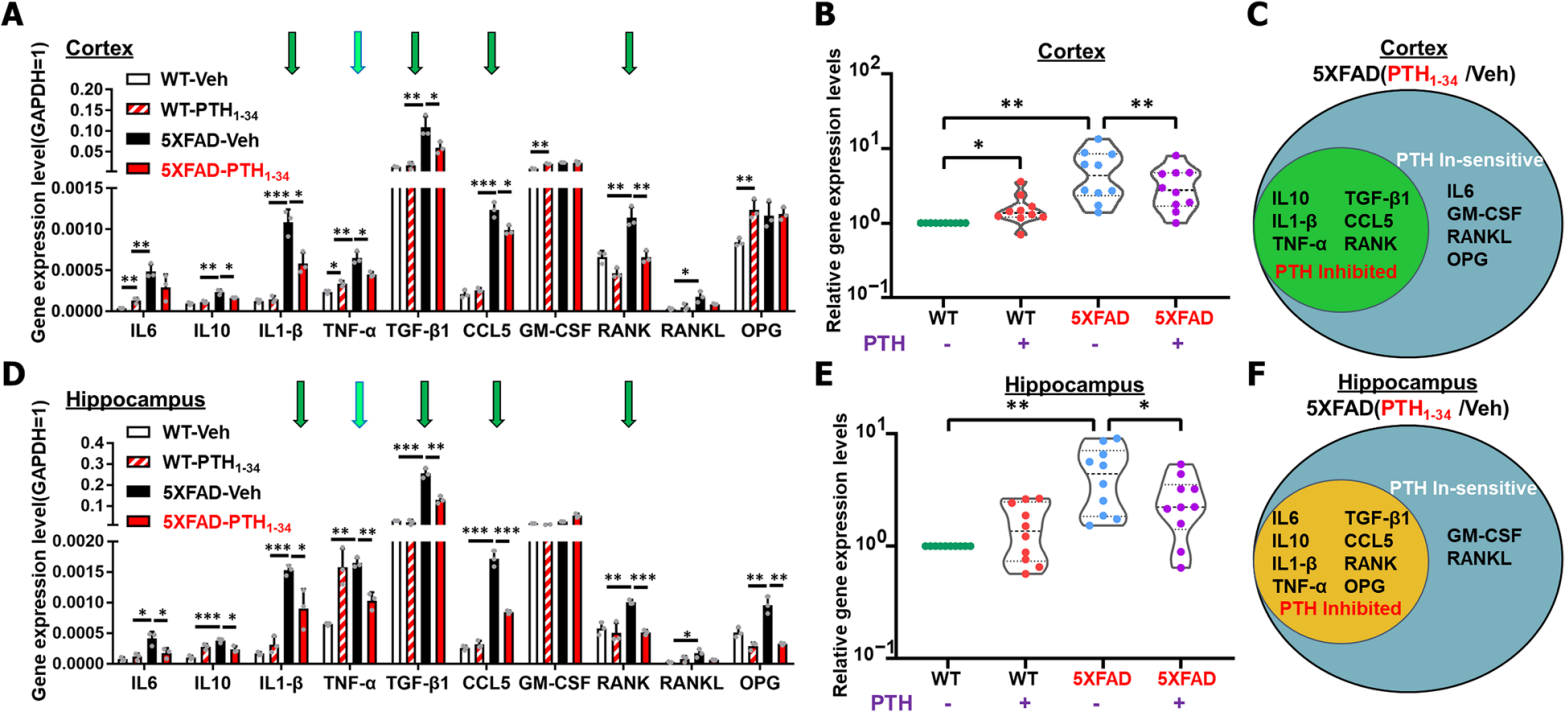
PTH_1-34_ diminishment of brain inflammation in 5XFAD mice. **A** Real-time PCR (RT-PCR) analysis of indicated gene expressions in the cortex of 6 ~ MO WT and 5XFAD female mice with PTH_1-34_ or Veh treatment. The level of GAPDH was normalized to 1, *n* = 3 per group. **B** Total quantification of relative gene expression levels in **A**. The level of WT group was normalized to 1, *n* = 10 gene per group. **C** The summary of altered genes in the 5XFAD-PTH_1-34_ treatment group compared with the 5XFAD-Veh group in cortex. **D** RT-PCR analysis of indicated gene expressions in the hippocampus of 6 ~ MO female mice, *n* = 3 per group. **E** Total quantification of relative gene expression levels in **D**. **F** The summary of altered genes in the 5XFAD-PTH_1-34_ treatment group compared with the 5XFAD-Veh group in hippocampus. All quantification data were presented as mean ± SD. *P < 0.05, **P < 0.01, ***P < 0.001, one-way ANOVA with Tukey’s multiple-comparison test was used

We then asked whether PTH_1-34_ treatments affected the expressions of proinflammatory cytokines and chemokines in 5XFAD brain. RT-qPCR analyzed ~ 10 genes’ expressions, which are either well-recognized proinflammatory cytokines (e.g., IL1β, IL6, TNFα, TGFβ) or cytokines involved in bone remodeling (e.g., RANKL, RANK, OPG, and GM-CSF), in 6 ~ MO old WT/5XFAD female brain (cortex and hippocampus) with PTH_1-34_ or vehicle treatments (Fig. 5A, D). While most of these factors were upregulated in 5XFAD brain (cortex and hippocampus), as compared with those of same-aged WT mice, these upregulated inflammatory cytokines or chemokines were largely brought down to nearly normal levels by PTH_1-34_ (Fig. 5A–B, D–E). Notice that while the majority of factors were downregulated by PTH_1-34_ treatments in both 5XFAD cortex and hippocampus, a few cytokines, largely factors involved in bone remodeling (e.g., GM-CSF, RANKL, and OPG in the cortex) occurred unaffected by PTH _1–34_ (Fig. 5C, F). This is in contrast to the effect of PTH in osteoblastic bone cells, where PTH is known to regulate RANKL and OPG expression [47, 48]. Taken together, these results suggest that PTH_1-34_ treatments reduce glial cell activation and consequent brain inflammation.

### PTH_1-34_ reduction of serum inflammatory cytokines

**Fig. 6:**
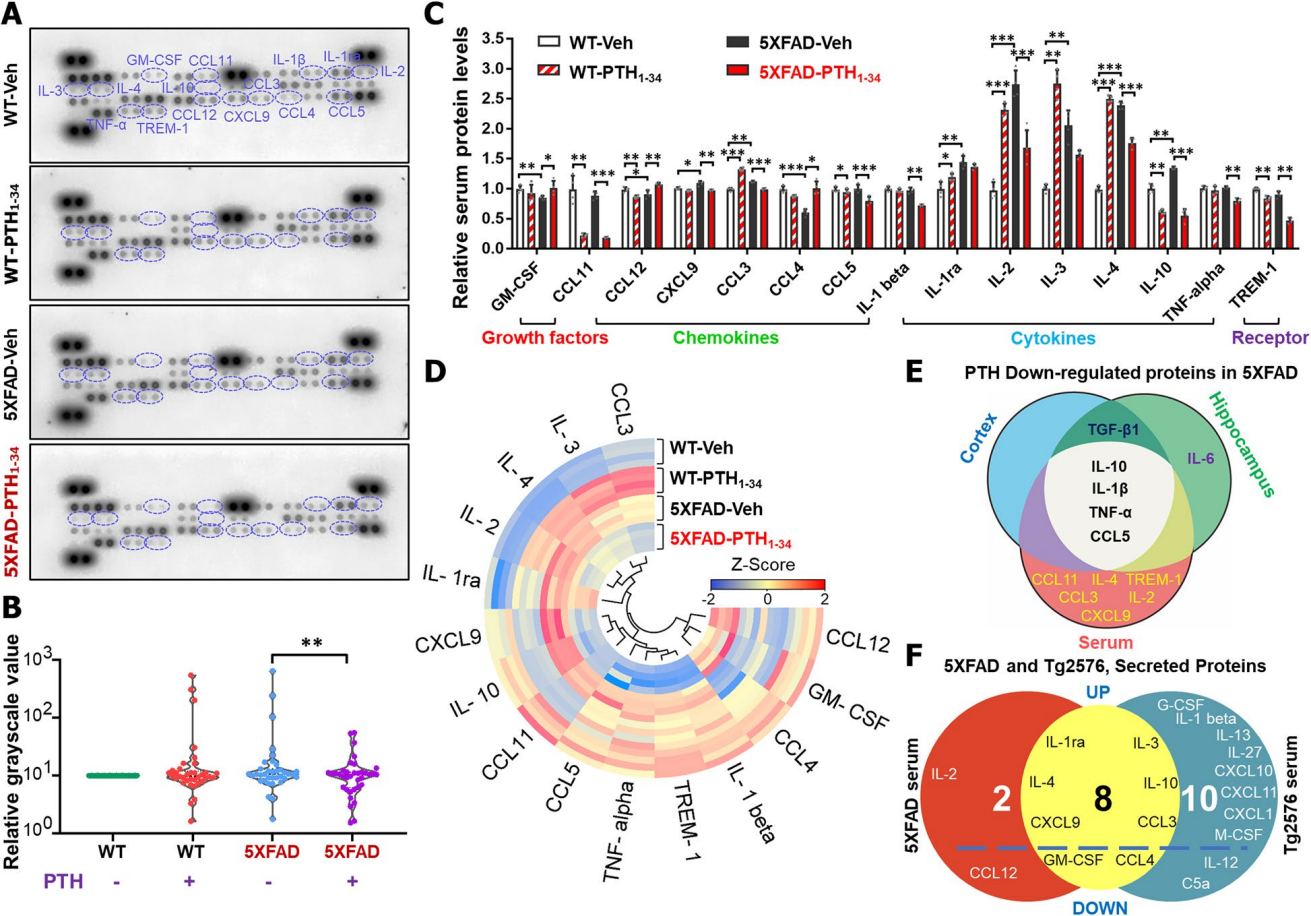
PTH_1-34_ reduction of serum inflammatory cytokines. **A** Representative images of serum L-Series label-multiplex antibody arrays of 6 ~ MO WT and 5XFAD female mice with PTH_1-34_ or Veh treatment. Proteins with changes are marked by dashed blue circles. **B** Total quantification analyses of the data in A. WT-Veh group were normalized to 1, *n* = 36 proteins per group. **C** Quantification of relative serum protein levels in **A**. The data showed those proteins that were significantly changed and arranged into different groups according to their characteristics. The data were presented as mean ± SD (*n* = 4 mice per group), *P < 0.05, **P < 0.01, ***P < 0.001, one-way ANOVA with Tukey’s multiple-comparison test. **D** Heat map of serum protein. *n* = 4, significant difference was set at *P* < 0.05. **E** Comparison of the PTH_1-34_ downregulated cytokines in serum of 5XFAD mice (downregulated cytokines in serum of PTH_1-34_ treated 5XFAD mice over 5XFAD control mice) to those detected in the cortex or hippocampus of 5XFAD-PTH_1-34_ mice. **F** Comparison between the 5XFAD with Tg2576 antibody array of secreted proteins in serum

To understand how PTH_1-34_ treatments affect brain inflammation in 5XFAD mice, we investigated PTH_1-34_’s effect on systemic inflammation, since PTH is a hormone with systemic effects, particularly in bone cells [17, 18]. Using multiplexed antibody-based arrays to screen for altered serum/plasma proteins in 5XFAD and WT female mice (6 ~ MO) with PTH_1-34_ or vehicle treatments, we found that among 40 factors, few (7/40) were elevated in serum samples from 5XFAD mice, as compared with that of WT mice (Fig. 6A–C), suggesting a weak systemic inflammation in 5XFAD mice. Notice that most of these elevated serum factors (e.g., IL-1rα, IL-2, IL-3, IL-4, IL-10, CXCL9, CCL3) in 5XFAD mice were all reduced by PTH_1-34_ treatments (Fig. 6C–D). Additionally, PTH_1-34_ reduced some serum inflammation-associated factors such as IL1β, TNFα, TREM-1, CCL11, and CCL5, which were comparable between WT and 5XFAD mice, and restored serum factor CCL4, which was decreased in 5XFAD mice (Fig. 6C–D). We compared the PTH_1-34_ downregulated cytokines in serum of 5XFAD mice with those in brain of 5XFAD, as shown in Fig. 6E. The results showed that 4 factors, IL-10, IL-1β, TNFα, and CCL5, were reduced by PTH_1-34_ treatments not only in the serum but also in the brain of 5XFAD, suggesting a certain relationship between systemic inflammation and brain inflammation (Fig. 6E). We further compared these changes in serum factors between 5XFAD and Tg2576 mice, another AD animal model expressing Swedish mutant APP ubiquitously [30, 49]. In contrast to that of 5XFAD mice, Tg2576 mice exhibited elevations in many (15/40) of these serum factors (Fig. 6F). These results demonstrate the systemic effect of PTH_1-34_, although systemic inflammation in 5XFAD mice seems to be weaker than in Tg2576 mice.

### PTH_1-34_ association with astrocytes in the brain

**Fig. 7:**
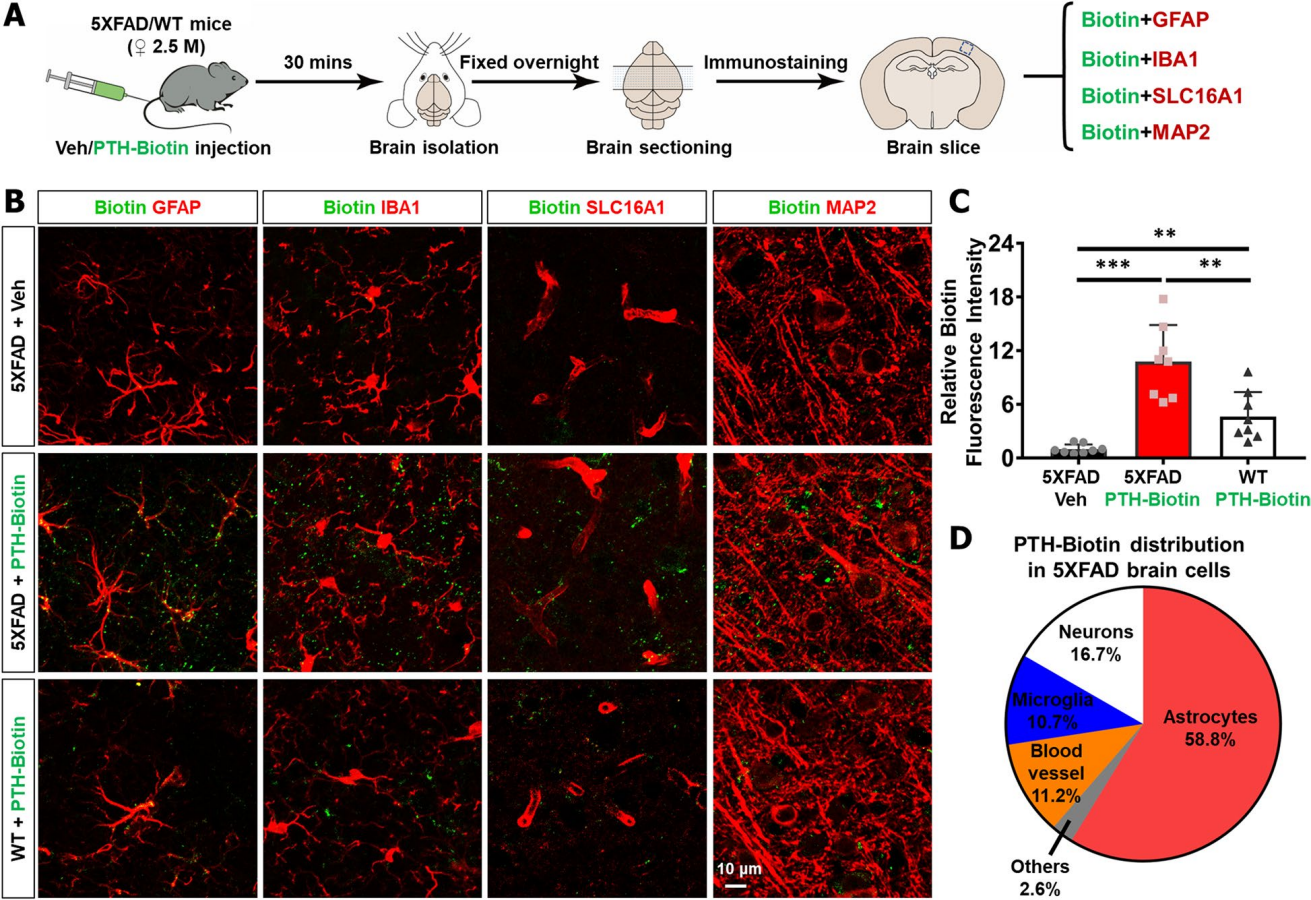
More PTH_1-34_ association with astrocytes in 5XFAD brain than that of WT control. **A** Schematic diagram of experimental design. 2.5 ~ MO WT and 5XFAD female mice were administered PTH_1-34_-Biotin (100 μg/100 μl) or vehicle (phosphate buffer, 100 μl) by tail-intravenous injection to detect PTH_1-34_ diffusion in vivo. Mice were sacrificed after 30 min for brain isolation and tissue sectioning, and immunofluorescence staining was used to analyze the distribution of PTH_1-34_-Biotin. **B** Representative images and high-magnification images of Biotin (green) co-immunostaining with GFAP, IBA1, SLC16A1, and MAP2 (red) respectively from 5XFAD + Veh, 5XFAD + PTH_1-34_-Biotin and WT + PTH_1-34_-Biotin female mice. All images were obtained from the cortex regions. Scale bars were indicated in the panel. **C** Quantification of relative Biotin fluorescence intensity in these three groups (mean ± SD; *n* = 8 mice per group). **P < 0.01, ***P < 0.001, one-way ANOVA with Kruskal–Wallis multiple-comparison test. **D** Quantification of PTH_1-34_-Biotin distribution in 5XFAD brain cells from B. The distribution of PTH_1-34_ in various cells was shown as a percentage, with a total proportion of 100%, derived from the mean value of data collected from all mice in the 5XFAD + PTH_1-34_-Biotin group

Given the significant effect of PTH_1-34_ in 5XFAD brain pathology, we wondered whether the injected PTH_1-34_ functioned directly in 5XFAD brain. To address this question, Biotin-conjugated PTH_1-34_ was administered into WT and 5XFAD female mice (~ 2.5 MO) by caudal vein injection, and mice were sacrificed 30 min after injection, as illustrated in Fig. 7A. Immunostaining analysis with biotin antibody showed more biotin signals in the brain of 5XFAD mice than in WT mice (Fig. 7B–C), implicating a BBB (blood–brain barrier) leakage in 5XFAD brain [3, 50] that causes more Biotin-PTH_1-34_ to enter the brain. Further co-immunostaining analysis showed that most Biotin-PTH_1-34_ signals were associated with GFAP^+^ astrocytes (~ 60%); ~ 10–17% of Biotin-PTH_1-34_ signals were in association with IBA1^+^microglia, SLC16A1^+^ blood vessels, and MAP2^+^ neurons, respectively (Fig. 7D). These results thus suggest that injected biotin-PTH_1-34_ can enter the brain through BBBs of 5XFAD mice and bind to astrocytes in large quantities.

The abundant association of Biotin-PTH_1-34_ with astrocytes leads us to ask whether this is due to the abundant expression of PTH receptor (PTH1R) in astrocytes. Indeed, scRNA-seq database showed selective PTH1R expression in astrocytes and endothelial cells in the brain. We further verified this view by RNA scope analysis (a higher resolution of in situ analysis), which showed co-distribution of PTH1R’s mRNAs largely with Glast1-mRNA^+^ astrocytes (Fig. S7C-D). Moreover, western blot analysis of lysates from primary cultured astrocytes demonstrated expression of PTH1R (Fig. S7A-B). Interestingly, the PTH1R protein level appeared to be slightly higher in astrocytes from 5XFAD mice than that of WT astrocytes (Fig. S7B-D). Together, these results support the view that injected PTH_1-34_ can travel to the brain and bind to its receptor in astrocytes in 5XFAD mice.

### PTH_1-34_ suppression of proinflammatory cytokines’ expression in 5XFAD astrocytes

To verify the view that PTH_1-34_ functions directly on astrocytes, we examined whether PTH_1-34_ could induce signaling and function in primary cultured astrocytes from WT and 5XFAD female pups (Fig. S8A). Interestingly, among PTH_1-34_ induced signaling pathways identified in osteoblasts (e.g., cAMP-driven phospho-CREB, phospho-AKT, and phospho-ERK1/2), PTH_1-34_ increases p-CREB, but has little effect on either p-AKT or p-ERK (Fig. S8B-E), suggesting that PTH_1-34_ induction of cAMP to p-CREB may be a key signaling pathway in astrocytes. Notice that a trend of increasing, but not significant, p-CREB was detected in 5XFAD astrocytes, as compared to WT astrocytes (Fig. S8C), which might be due to a slight increase in the expression of PTH1R in 5XFAD astrocytes. These results thus support the view that PTH_1-34_ functions directly on astrocytes.

We further tested this view by examining PTH_1-34_’s effect on the expression of cytokines and chemokines in astrocytes. Cultured astrocytes from WT and 5XFAD female pups were treated with vehicle or PTH_1-34_ for 24 h, and then subjected to RT-qPCR analysis, as illustrated in Fig. S9A. Increased levels of cytokines or chemokines (e.g., TNF-α, TGF-β1, CCL5, GM-CSF, and RANK) were detected in 5XFAD astrocytes, as compared with those in WT astrocytes (Fig. S9B-D), consistent with multiple literature reports of a higher basal inflammatory state in 5XFAD cells [51, 52]. Nearly all these factors (except GM-CSF) were reduced by PTH_1-34_ treatments (Fig. S9B, E). We compared these changes with PTH_1-34_ downregulated genes in the 5XFAD brain (cortex and hippocampus). As illustrated in Fig. S9F, 4 factors, TGF-β1, CCL5, RANK, and TNFα, were reduced by PTH_1-34_ treatments not only in cultured 5XFAD astrocytes but also in 5XFAD brain. These results provide additional support for PTH_1-34_ functioning in astrocytes, where it suppresses the expression of inflammatory cytokines or chemokines.

### PTH_1-34_ decrement of senescence-like astrocytes from 5XFAD mice

**Fig. 8:**
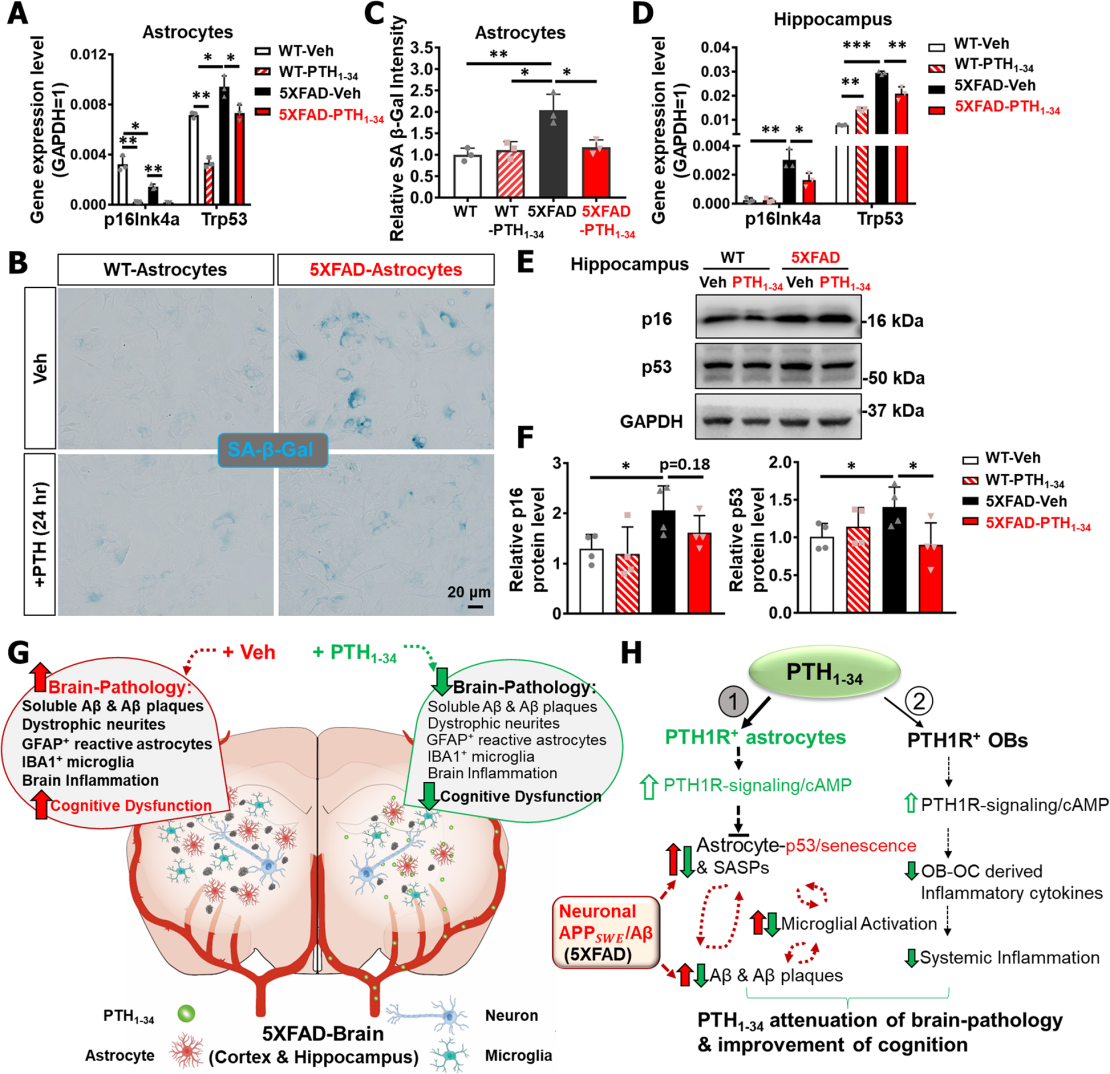
PTH_1-34_ decrement of senescence-like astrocytes from 5XFAD mice. **A** RT-PCR analysis of senescence-related gene expression level in the cultured astrocytes with PTH_1-34_ or Veh treatment from WT and 5XFAD P3 female pups. GAPDH expression level was normalized to 1, *n* = 3 independent experiments. **B** SA-β-gal staining of cultured WT and 5XFAD astrocytes with PTH_1-34_ or Veh treatment. Scale bars as indicated in the panel. **C** Quantification of relative SA-β-gal + cell intensity in B (mean ± SD; *n* = 3 independent experiments). **D** RT-PCR analysis of senescence-related gene expression level in the hippocampus from 6 ~ MO WT or 5XFAD female mice with PTH_1-34_ or Veh treatment. GAPDH expression level was normalized to 1 (*n* = 3). **E** Western blot analysis of indicated protein expression in homogenates of hippocampus of 6 ~ MO WT and 5XFAD female mice with PTH_1-34_ or Veh treatment. **F** Quantification of relative protein level in **E**, *n* = 4 mice per group. All quantification data were presented as mean ± SD. *P < 0.05, **P < 0.01, one-way ANOVA with Tukey’s multiple-comparison test was used. **G** Summary of AD brain pathology and cognitive function differences in 5XFAD mice with Veh or PTH_1-34_ intermittent treatment. **H** Illustration of the working model

It is known that increased proinflammatory cytokines and chemokines in the brain/astrocytes of 5XFAD mice exhibit features of SASPs (senescence-associated secretory phenotypes) [14, 53]. Given reports of astrocyte senescence in the brains of AD patients [54], we asked whether astrocytes from 5XFAD mice showed cellular senescence features and whether this event was affected by PTH_1-34_ treatments. To this end, cultured astrocytes from WT and 5XFAD female pups were treated with PTH_1-34_ or vehicle for 24 h and examined the expression of senescence markers, including p16^Ink4a^, p53, and SA-β-gal (senescence associated β-gal) [55, 56]. RT-PCR analysis showed increased p53, but not p16^Ink4a^, in 5XFAD astrocytes, which was diminished by PTH_1-34_ (Fig. 8A), implicating possible senescence in 5XFAD astrocytes. This view was further supported by SA-β-gal analysis, which showed increased SA-β-Gal^+^ astrocytes from 5XFAD mice, and attenuated by PTH_1-34_ treatments (Fig. 8B–C). Given the above data, we subsequently tested this view in vivo. RT-PCR analysis of both p16^Ink4a^ and p53 transcripts showed a clear increase in the 5XFAD hippocampus and a great reduction after PTH_1-34_ treatment, compared with WT group (Fig. 8D). In line with these results, western blot analysis showed an increase in p16^Ink4a^ and p53 in the female 5XFAD hippocampus homogenates, with p53 reduced by PTH_1-34_ treatments (Fig. 8E–F). Together, these results suggest that p53 may be a driver of astrocyte senescence in the brain of 5XFAD mice, which is attenuated by PTH_1-34_, implicating PTH_1-34_ decrement of astrocyte senescence in 5XFAD mice as a potential cellular mechanism for its beneficial effects.

## Discussion

AD is the most common form of dementia and there is currently no effective therapy. Interestingly, in addition to brain atrophy and memory loss in the early stages of disease, AD patients often have lower bone mineral density or osteoporosis [7–10]. PTH_1-34_ is clinically indicated for the treatment of osteoporosis [16–18]. While there have been reports implicating low or high levels of PTH in neurodegenerative diseases including AD [15, 20, 21], it remains largely unclear if PTH_1-34_ treatment has any effect on AD brain pathology and/or AD progression. Here, using 5XFAD mice with PTH_1-34_ intermittent treatments, we found that PTH_1-34_ functions not only in bone cells, but also in brain cells (largely astrocytes), where PTH_1-34_ suppresses astrocyte senescence and expression of proinflammatory cytokines, resulting in reductions in glial cell activation, dystrophic neurites, Aβ levels, and Aβ deposition, and thus improving the cognitive function of 5XFAD mice. These observations lead us to propose a working model depicted in Fig. 8G–H.

It is known that PTH is a polypeptide hormone secreted by cells of the parathyroid gland. Its analogs, such as PTH_1-34_, have been used as a therapy for osteoporosis through its intermittent treatment [17, 18]. It is of interest to notice reports that abnormal PTH levels may be associated with neuronal calcium imbalance, cognitive decline, and neuroinflammation [22, 23, 25, 27]. Considering these reports and in light of the association of AD with osteoporosis, we wondered whether PTH_1-34_ treatments could have an effect on AD, in addition to osteoporosis. Indeed, several lines of evidence suggest that this is precisely the case. PTH_1-34_ intermittent treatments not only increase trabecular bone volumes and bone formation (Fig. 1) but also decelerate Aβ-associated brain pathology in 5XFAD mice (Fig. 8G). The Aβ levels, Aβ deposition (Fig. 3), dystrophic neurites (Fig. S3), reactive microglia and astrocytes (Fig. S4A-B and Fig. 4), and brain inflammatory (Fig. 5) were all diminished by PTH_1-34_ treatments in 5XFAD mice, and the cognitive functions (Fig. 2) in 5XFAD mice (in particular, female) were also improved. Notably, the therapeutic effect of PTH_1-34_ was enhanced in female mice, as amyloid deposition levels in 5XFAD female mice after PTH_1-34_ treatment were reduced by a greater proportion than in male mice (Fig. 3D–L). PTH_1-34_ treatment brings the level of Aβ deposition in female 5XFAD mice down to the level of untreated 5xFAD male mice or even lower. Such a female-dependent PTH_1-34_ effect may be due to the earlier and more severe onset of amyloid pathology in female 5XFAD mice [29, 39, 40].

How do PTH_1-34_ treatments attenuate brain pathology in 5XFAD mice? As illustrated in Fig. 8H, we speculate that PTH_1-34_, by binding to its receptor PTH1R in bone osteoblasts and brain astrocytes, suppresses cellular senescence, systemic inflammation, and brain inflammation, thereby alleviating AD pathology in 5XFAD mice. This speculation is in light of the following observations. First, PTH1R is expressed not only in osteoblasts [57, 58] but also in astrocytes (Fig. S7). Second, PTH_1-34_ may affect cytokine expression not only in osteoblasts (e.g., RANKL, RANK, OPG, TGF-β1, IL-6, TNF-α, etc.) [47, 48, 59, 60], but also in astrocytes (Fig. S9). Third, PTH_1-34_ appears to act as a senolytic drug, with a recent report showing that PTH protects bone from age-induced bone loss by protecting osteoblasts/osteocytes from oxidative stress-induced cell death and senescence [61]. In our study, PTH_1-34_ suppressed astrocyte senescence (Fig. 8A–C), an event that induces SASPs and inflammatory cytokine expression [53, 54]. Although PTHrP (Parathyroid hormone-related protein) has been reported in neurons in normal mouse brains and can be induced in reactive astrocytes in inflamed brains or after brain injury [62], our study shows that exogenous PTH_1-34_ can enter the brain and bind to astrocytes. It is important to note that the Biotin-PTH_1-34_ signals were detectable in the brains of WT mice after its injection, suggesting the possibility of “PTH_1-34_ infiltration into the brain” of WT mice under normal BBB condition. However, more Biotin-PTH_1-34_ enters the brain of 5XFAD mice, as compared to that of WT controls (Fig. 7B–C). This may be due to the BBB impairment in 5XFAD mice [3, 50]. It is noteworthy that the expression of PTH1R in brain has been detected in other species, including zebrafish, chicken, or xenopus [20, 57, 63]. We believe that astrocytes may be the dominant cell type in which PTH_1-34_ functions in the brain, as the majority of injected biotin-PTH_1-34_, is associated with astrocytes (Fig. 7D); astrocytes express abundant PTH1R (Fig. S7), and PTH_1-34_ could induce signaling in primary cultured astrocytes (Fig. S8).

Is PTH_1-34_ a senolytic-like drug? The increase of senescent cells in aged tissues is thought to cause a functional decline in homeostasis and integrity and is associated with diminished response to physiological conditions under stress [64, 65]. Cellular senescence is thus tightly linked to brain aging and various degenerative diseases, including AD and osteoporosis. Interestingly, it has been noted that astrocytes in the brain of AD patients undergo senescence [54, 66] and that the use of senolytic drugs can attenuate AD-relevant brain pathology in AD animal models [67, 68]. PTH_1-34_ appears to be a senolytic-like drug that not only protects osteoblasts/osteocytes from oxidative stress-induced cell death and senescence [61], but also reduces the senescence phenotype in astrocytes (Fig. 8A–C). We detected increased senescence markers (e.g., SA-β-gal, and 53) in cultured primary astrocytes from 5XFAD mice. Upon PTH_1-34_ treatments, these senescence markers in astrocytes were reduced, as were SASP-like cytokines in 5XFAD astrocytes and 5XFAD brain (Fig. 8A–C). These observations thus support the view for PTH_1-34_ to act as a senolytic-like drug.

Astrocytes, the most abundant glial cells in brain, play an important role in AD development. During the progression of AD, amyloid deposition leads to brain inflammation caused by activation of astrocytes and microglia, further promoting the pathological manifestations, including synaptic dysfunction, neuroinflammation, and the accumulation of amyloid plaques in a vicious cycle [69–71]. Our results of decreased total GFAP^+^ astrocytes (Fig. 4) and brain inflammatory cytokines (Fig. 5) in PTH_1-34_ treated 5XFAD mice suggest a role of PTH_1-34_ in suppressing astrocyte activation. This view is also supported by the results obtained in cultured astrocytes, which showed downregulation of cytokines and chemokines (TGF-β1, TNF-α, CCL5, RANK) in 5XFAD astrocytes treated with PTH_1-34_ (Fig. S9). Moreover, this view is consistent with multiple literature reports that eliminating the inflammatory overload states in brain improves the pathology of neurodegenerative diseases [72, 73].

In addition to its function in astrocytes, PTH_1-34_’s effect on osteoblasts and systemic inflammation may also underlie its beneficial roles in inhibiting AD-related brain pathology. Evidence suggests that systemic inflammation may influence local inflammation in the diseased brain, leading to excessive synthesis of inflammatory cytokines and other mediators in the brain, which in turn may contribute to the outcome or progression of chronic neurodegenerative disease [74]. Chronic inflammation is a common risk factor for both osteoporosis and AD [75–77]. Systemic inflammation, including a few elevated serum inflammatory factors, was nearly abolished by PTH_1-34_ treatments in 5XFAD mice (Fig. 6A–D), suggesting a systemic effect of PTH_1-34_. However, a comparison of serum inflammation factors between the 5XFAD and Tg2576 mice showed a weaker systemic inflammation in 5XFAD mice than that in the Tg2576 AD animal model (Fig. 6F). This may be due to the differential mutant APP expression pattern between the two AD mouse lines. While Tg2576 mice express mutant APP ubiquitously, including in bone cells, under the control of prion promoter [28], 5XFAD mice express mutant APP under the control of Thy1 promoter, thus driving the expression of mutant APP largely in neurons and immune cells [29, 78].

## Conclusions

In summary, this study suggests the anti-senile function of PTH_1-34_ intermittent treatments in 5XFAD mice, exhibiting PTH_1-34_ induced reductions in Aβ pathology, systemic and brain inflammation, and PTH_1-34_ improvement in cognitive function in 5XFAD mice and implicating PTH_1-34_’s potential therapeutic effects for patients with not only osteoporosis but also AD. At cellular level, PTH_1-34_ binds to PTH1R not only in osteoblasts but also in astrocytes, suppressing cellular senescence, systemic inflammation, and brain inflammation. We hope to further investigate the molecular underlying mechanisms of PTH_1-34_’s function in the brain in future.

## Supplementary Information

**Additional file 1: **This file contains all the Supplemental Figures.

**Additional file 2: Supplemental file 1.** Two-way ANOVA analysis results.

**Additional file 3: Supplemental file 2. **Full western blots.

## Data Availability

The datasets generated and/or analyzed during the current study are included in this published article and its supplementary information files.

## Abbreviations

PTH: Parathyroid hormone
AD: Alzheimer’s disease
Aβ: β-Amyloid
BMD: Bone mineral density
WT: Wild type
APP: Amyloid beta precursor protein
Veh: Vehicle
µCT: Micro CT
BV: Bone volume
TV: Total volume
Tb.N: Trabecular bone number
Tb.Th: Trabecular bone thickness
Tb.Sp: Trabecular bone space
PYD: Pyridinoline
NOR: Novel object recognition
MWM: Morris water maze
PBS: Phosphate buffer
PFA: Paraformaldehyde
Thio-S: Thioflavin S
ROI: Region of interest
SA-β-gal: Senescence associated β-gal
SASPs: Senescence associated secretory phenotypes
DAM: Degeneration associated microglia
BBB: Blood-brain barrier
PTH1R: Parathyroid hormone 1 receptor
