# Supplementary material for: Attenuation of Alzheimer’s brain pathology in 5XFAD mice by PTH1-34, a peptide of parathyroid hormone
Source: Alzheimers Res Ther. 2023 Mar 14;15:53. doi: 10.1186/s13195-023-01202-z (PMC10012528; doi:10.1186/s13195-023-01202-z)
Supplement: Supplementary file 1 — Additional file 1: This file contains all the Supplemental Figures. [file 13195_2023_1202_MOESM1_ESM.pdf]

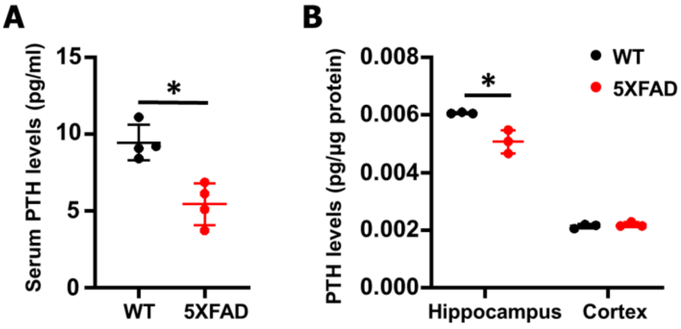

Fig. S1. Decreased PTH in 5XFAD mice. (A) Serum PTH levels in 6-MO WT and 5xFAD female mice. **(B)** PTH levels in cortex and hippocampus of 6-MO WT and 5XFAD female mice. The PTH levels were measured by Elisa. The values presented are means ±SD (*n* = 3 to 4). P values obtained by unpaired two-tailed t-test. * *P* < 0.05, significant difference.

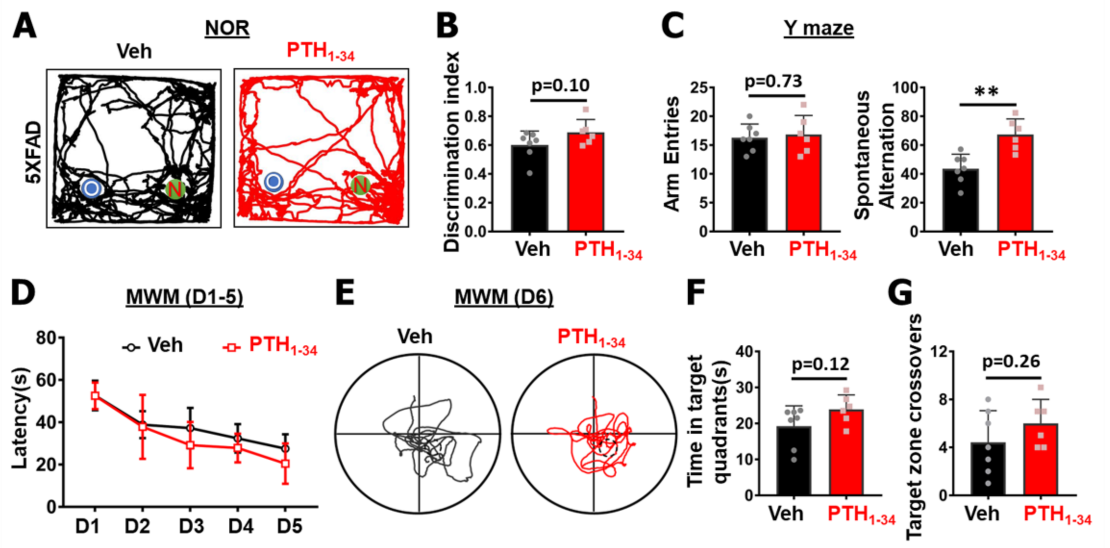

Fig S2. Little to weak improvement of the cognitive functions in 5XFAD male mice by PTH$_{1\text{-}34}$. 5XFAD male mice with Veh or PTH$_{1\text{-}34}$ treatment were subject to behavioral tests from 5~MO of age, as was the case for 5xFAD female mice. The results shown in this figure were for male mice. **(A, B)** NOR: Representative tracing images(A), and quantification of the discrimination index of NOR(B) were shown. **(C)** Y maze: Quantifications of the total arm entries and spontaneous alternation in Y maze. **(D-G)** MWM: The latencies to reach the hidden platform during the training period(D), the representative tracing images(E), quantification of time in the target quadrant(F), and target zone crossovers(G) on the testing day. All quantification data were shown as mean ± SD (n = 7 mice for 5XFAD-Veh group and n = 6 mice for 5XFAD-PTH$_{1\text{-}34}$ group). *$p < 0.05$, **$p < 0.01$, two-way ANOVA with Sidak's multiple comparisons test was used in D, and Student's t-test was used in B, C, F, and G.

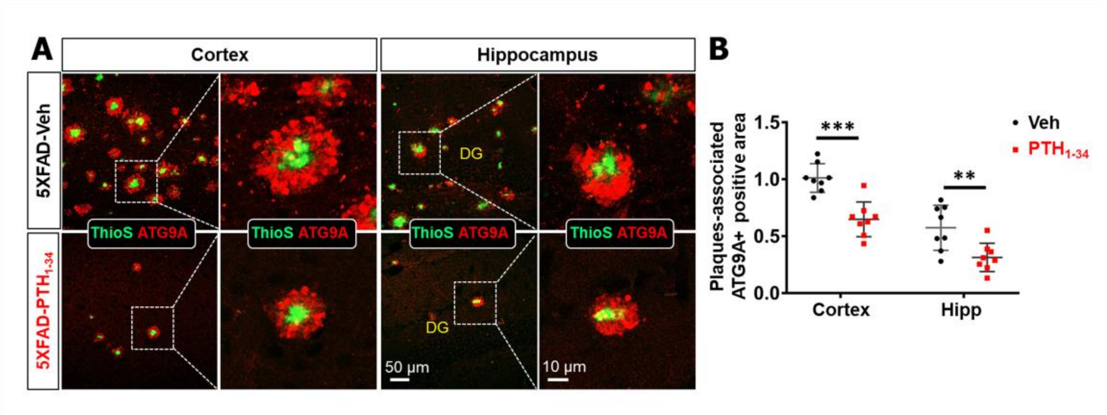

Fig S3. PTH$_{1-34}$ reduction of plague-associated dystrophic neurites. (A) Representative images of co-immunostaining with ThioS (green) and ATG9A (red) of the cortex and hippocampal sections from 6~MO 5XFAD-Veh and 5XFAD-PTH$_{1-34}$ female mice. **(B)** Quantification of Dystrophic neurites (ATG9A+ positive area) in A. Scale bars were indicated in the panel. Quantification data were presented as mean ± SD (n = 8 for per group). *$p < 0.05$, **$p < 0.01$, ***$p < 0.001$. Student's t-test.

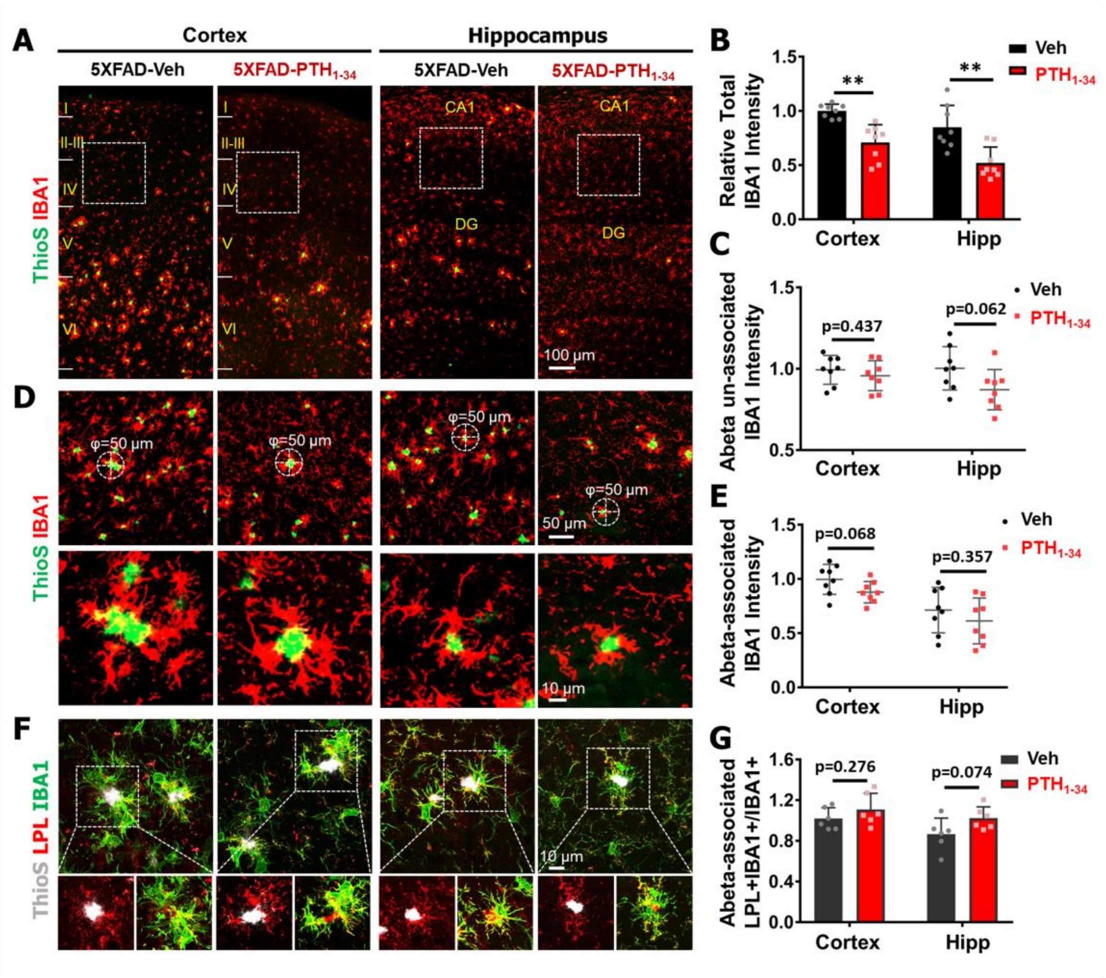

Fig S4. Little to no change in plaque-associated microglial cells in 5XFAD mice treated with PTH$_{1-34}$. (A) Representative images of co-immunostaining with ThioS (green) and IBA1 (red) of cortex and hippocampus from 6~MO 5XFAD-Veh and 5XFAD-PTH$_{1-34}$ female mice. **(B)** Quantification of the relative total IBA1 fluorescence intensity in A. **(C)** Quantification of Abeta un-associated IBA1 fluorescence intensity in A. The region used for quantification (no Aβ deposition area) were marked by white dashed squares, which were shown in A. **(D)** Representative images and high-magnification images in Aβ deposition regions of co-immunostaining with ThioS (green) and IBA1 (red) of cortex and hippocampus from 6-MO 5XFAD-Veh and 5XFAD-PTH$_{1-34}$ female mice. **(E)** Quantification of Abeta-associated IBA1 fluorescence intensity in D. The Abeta-associated IBA1 fluorescence intensity was defined by the intensity of IBA1 positive microglia in a plaque-centered circle within 50 μm in diameter (marked by dashed white circles). n = 8 mice per group. **(F)** Representative images of co-immunostaining with ThioS (white), IBA1 (green), and LPL(red) of cortical and hippocampal sections from 6-MO 5XFAD female mice with Veh/PTH$_{1-34}$ treatments. **(G)** Quantification of Abeta-associated LPL+IBA1+/IBA1+ in F (n = 6 mice per group). Scale bars as indicated in each panel. All quantification data were presented as mean ± SD. $^{*}p < 0.05$, $^{**}p < 0.01$, $^{***}p < 0.001$, Student's t-test.

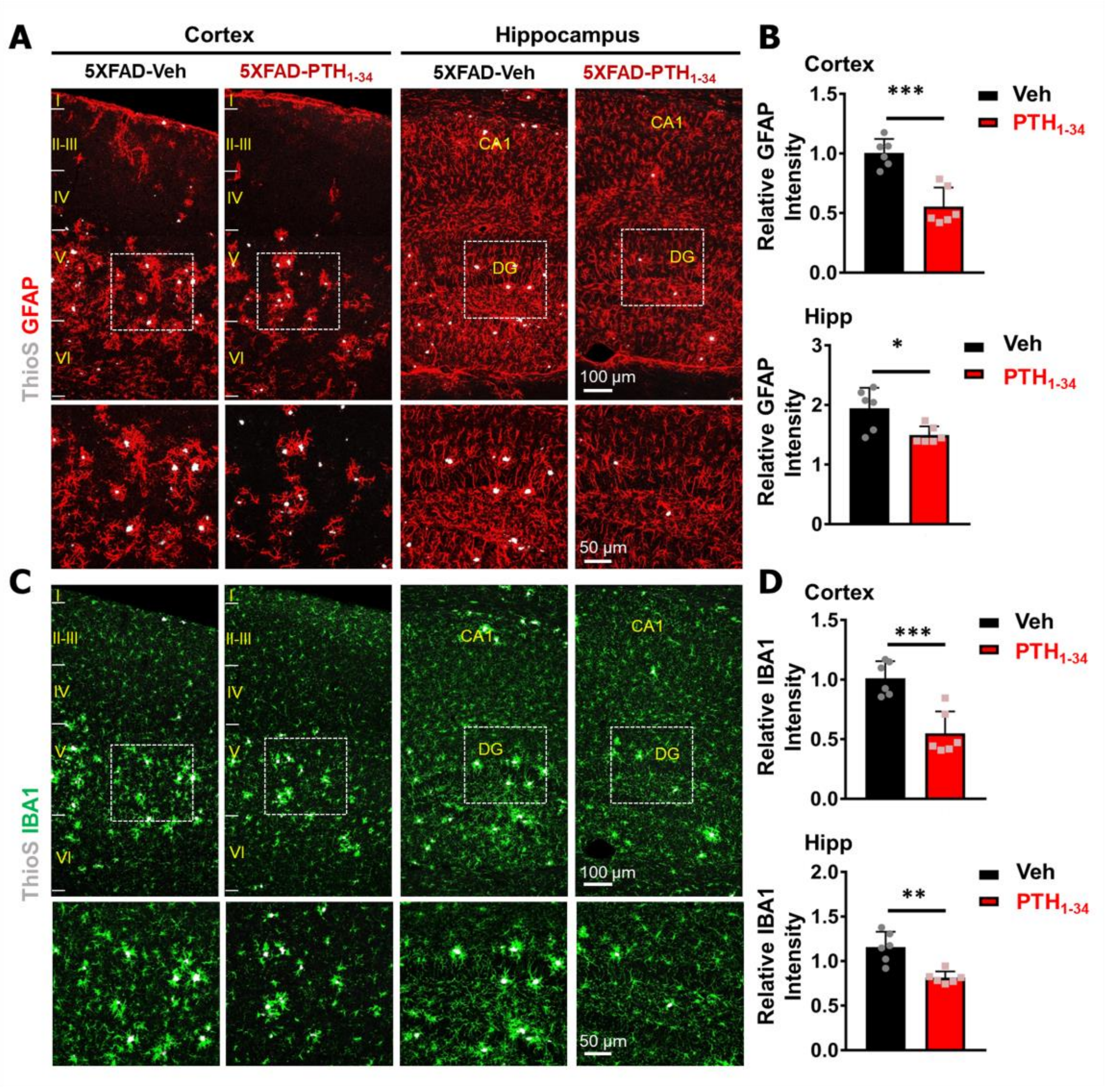

Fig S5. Significant reduction of glial cells in PTH_{1-34} treated 5XFAD male mice. (A) Representative overall images and high-magnification images in the Aβ deposition region of co-immunostaining with ThioS (green) and GFAP (red) of cortex and hippocampus from 6~MO 5XFAD-Veh and 5XFAD-PTH_{1-34} male mice. **(B)** Quantification of total relative GFAP fluorescence intensity of cortex and hippocampus in A. **(C)** Representative overall images and high-magnification images in the Aβ deposition region of co-immunostaining with ThioS (green) and IBA1 (green) of cortex and hippocampus from 6~MO 5XFAD-Veh and 5XFAD-PTH_{1-34} male mice. **(D)** Quantification of total relative IBA1 fluorescence intensity in C. Scale bars were indicated in each panel. All quantification data were presented as mean ± SD (n = 6 mice per group). *$p < 0.05$, **$p < 0.01$, ***$p < 0.001$, Student's t-test was used.

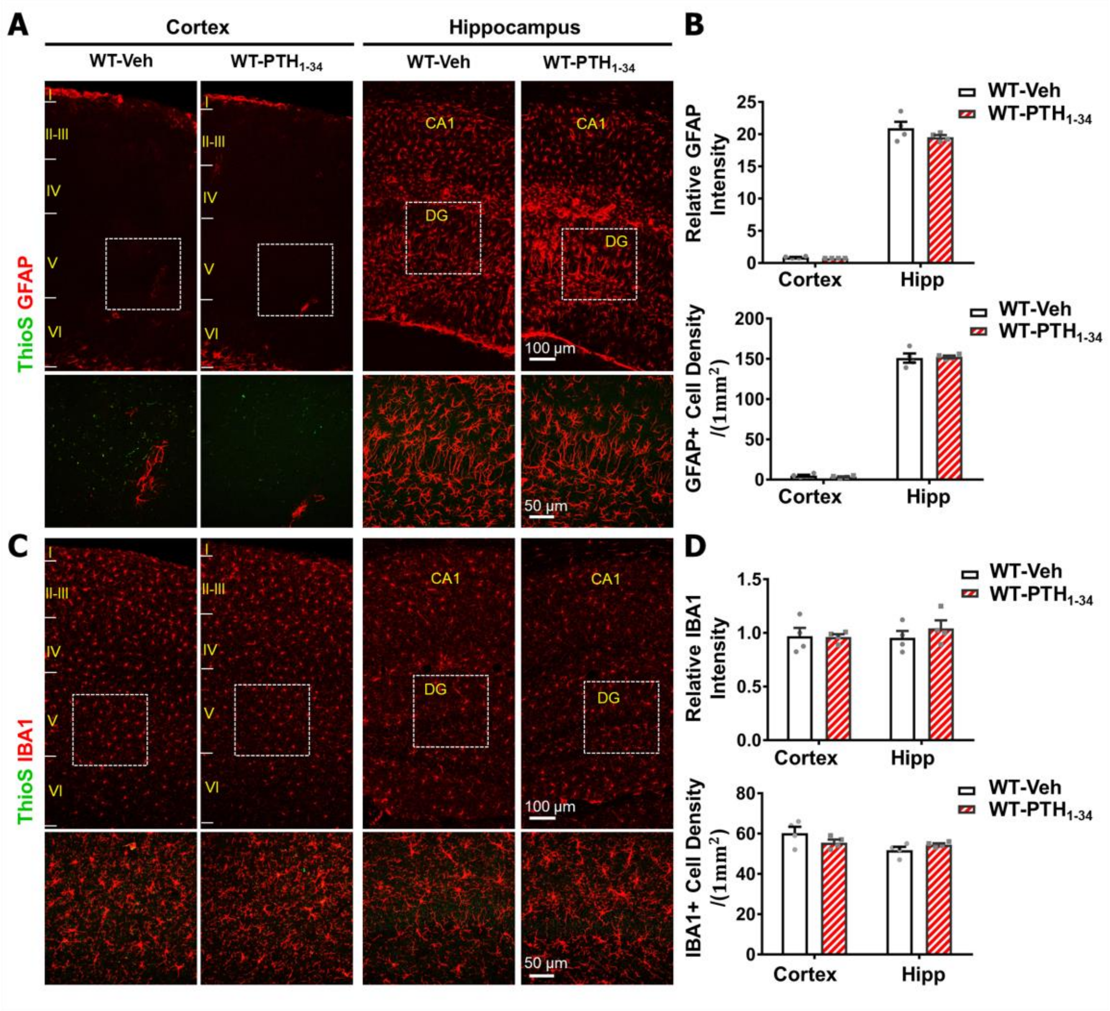

Fig S6. Little to no effect on astrocytes and microglia in the brains of WT mice treated with PTH$_{1-34}$. (A) Representative images of co-immunostaining with ThioS (green) and GFAP (red) of cortex and hippocampus from 6~MO WT-Veh and WT-PTH$_{1-34}$ female mice. **(B)** Quantification of relative GFAP fluorescence intensity and GFAP+ cell density in A. Cell density was defined by the number of positive cells per unit area (1 mm^2). **(C)** Representative images of co-immunostaining with ThioS (green) and IBA1 (red) respectively of cortex and hippocampus from 6~MO WT-Veh and WT-PTH$_{1-34}$ female mice. **(D)** Quantification of relative IBA1 fluorescence intensity and IBA1+ cell density in C. Scale bars were indicated in the panel. Quantification data were presented as mean ± SD (*n* = 4 for per group), Student's t-test.

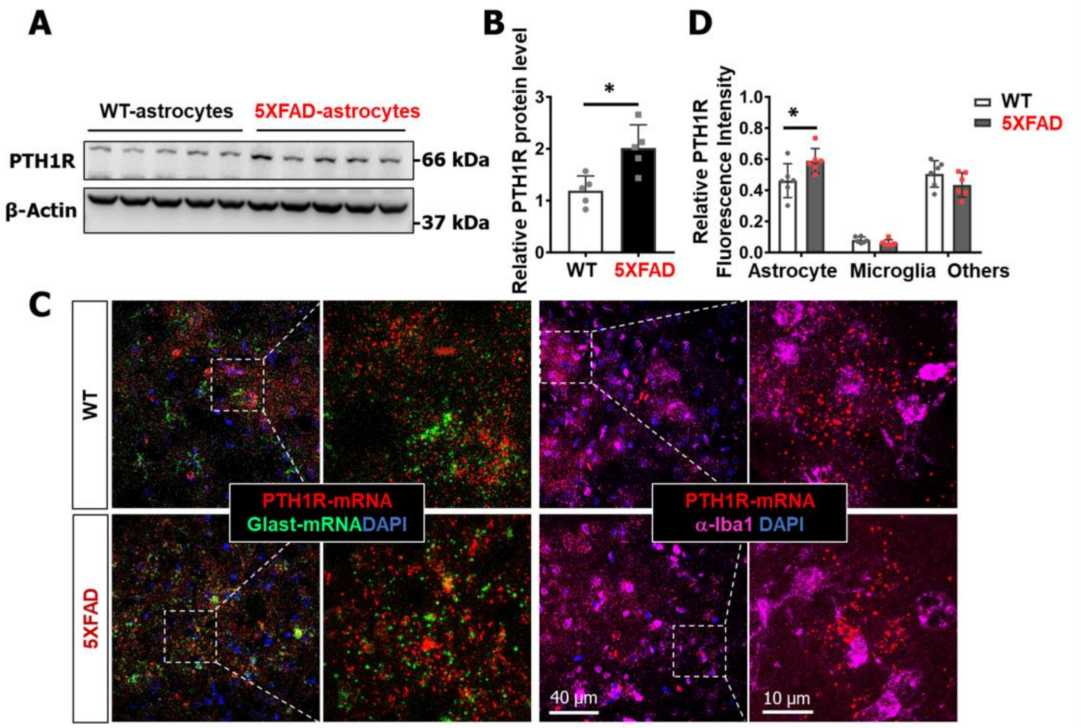

Fig S7. PTH1R expression in astrocytes. (A) Representative Western blots using antibodies against PTH1R in homogenates of cultured astrocytes from WT and 5XFAD pups. β-Actin was used as a loading control. **(B)** Quantification of PTH1R protein level in A (mean ± SD; n = 5). *p < 0.05, Student's t-test. **(C)** Representative images of RNA-Scope staining with PTH1R-mRNA (red), Glast-mRNA (green), IBA1 antibody (magenta), and DAPI (blue) of cortex sections from 6~MO WT and 5XFAD female mice. High-magnification images, marked by dashed squares, were shown in the right panels. Scale bars as indicated in each panel. **(D)** Quantification analyses of PTH1R fluorescence distribution in C. The distribution of PTH1R in different cells was shown as a ratio, with a total ratio of 1. *p < 0.05, mean ± SD, n =6 mice per group, two-way ANOVA with Sidak's multiple comparisons test was used.

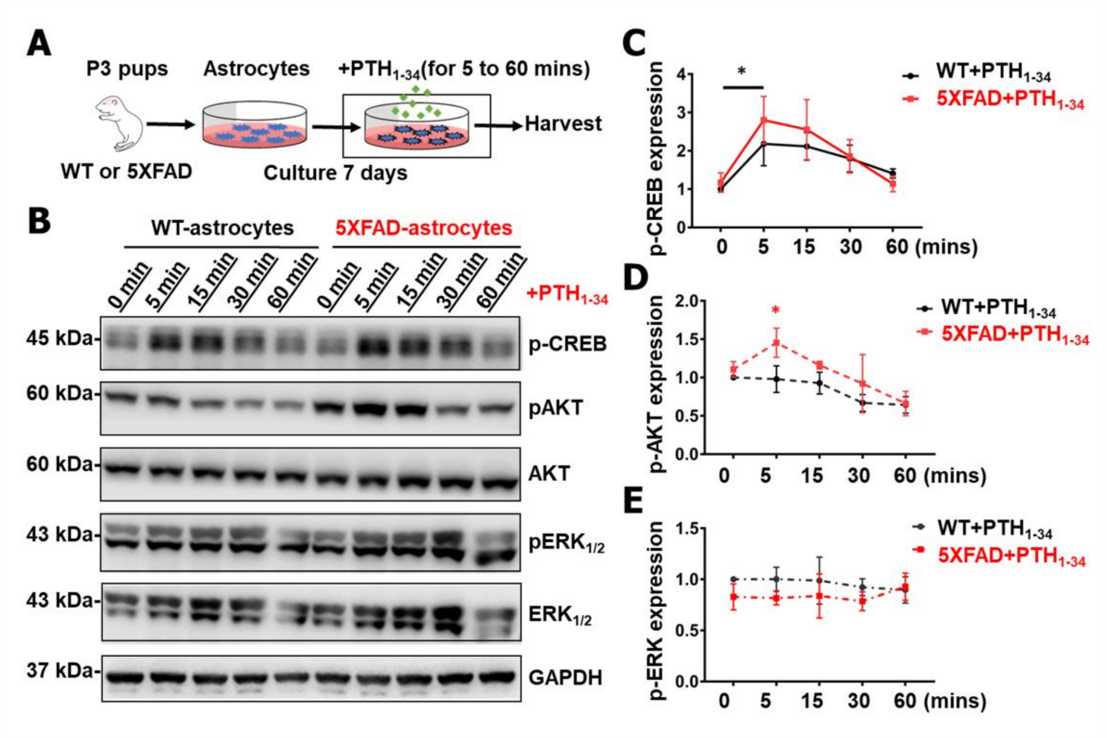

Fig S8. $PTH_{1\text{-}34}$ induction of signaling in astrocytes. (A) Illustration of astrocyte culture from WT or 5XFAD pups and the $PTH_{1\text{-}34}$ treatment. Cells were collected at different times of treatment and different pathway proteins were detected. **(B)** Western blot analysis of indicated protein expression in cultured astrocytes with different treatment times. GAPDH was used as a loading control. **(C-E)** Quantification analyses of the data in B. The curves of p-CREB protein level (C), p-AKT protein level (D), and p-ERK protein level (E) over time are shown. The level of WT group with 0 min $PTH_{1\text{-}34}$ treatment was normalized to 1. The data were presented as mean ± SD (*n* =3 independent experiments), *$p < 0.05$, and two-way ANOVA with Tukey's multiple-comparison test was used.

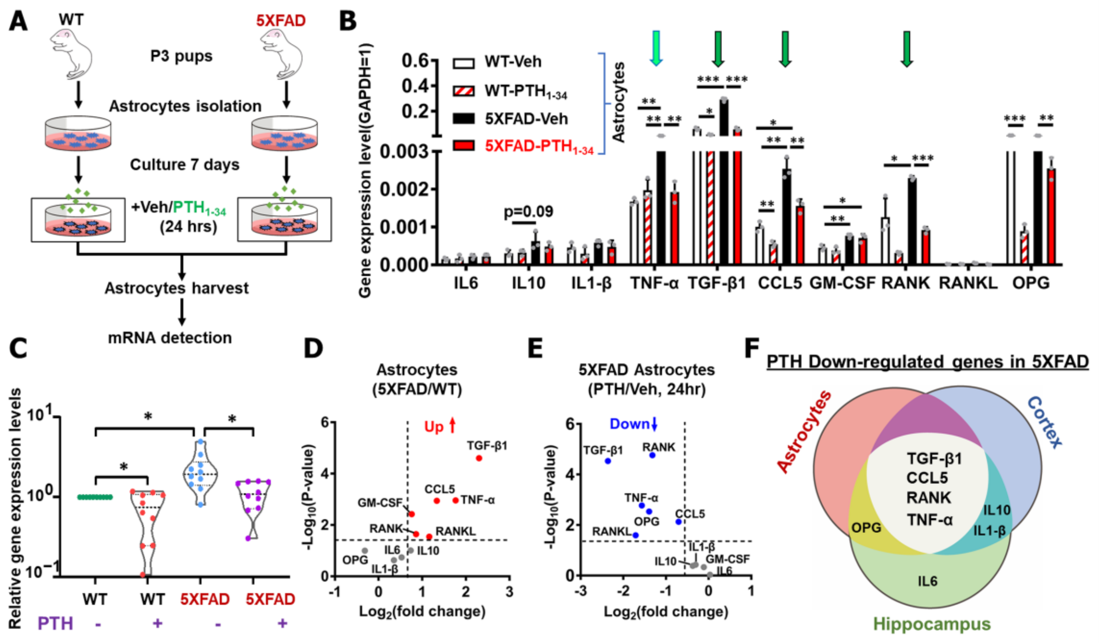

Fig S9. PTH$_{1\text{-}34}$ suppression of proinflammatory cytokine expression in 5XFAD astrocytes. **(A)** Schematic of the experimental design. Primary astrocytes derived from the brains of P3 WT and 5XFAD pups were treated with PTH$_{1\text{-}34}$ or vehicle for 24 h for gene expression detection. **(B)** RT-PCR analysis of indicated gene expressions in the cultured astrocytes of four groups. The expression of GAPDH was normalized to 1, *n*=3 independent experiments, $^{*}p < 0.05$, $^{**}p < 0.01$, $^{***}p < 0.001$, one-way ANOVA with Tukey's multiple-comparison test. **(C)** Total quantification of relative gene expression levels in B. The level of WT-Veh group was normalized to 1. $^{*}p < 0.05$, mean ± SD, one-way ANOVA test. **(D)** Volcano plots analysis of altered gene expression level between 5XFAD and WT astrocytes. **(E)** Volcano plots analysis of 5XFAD astrocytes gene expression alteration in the PTH$_{1\text{-}34}$ treatment group. The red dots showed up-regulated genes and the blue dots showed down-regulated genes. **(F)** Comparison of the changes (down-regulated genes in PTH$_{1\text{-}34}$ treated 5XFAD astrocytes over 5XFAD control astrocytes) to those detected in the cortex or hippocampus of 5XFAD-PTH$_{1\text{-}34}$ mice.
