# Supplementary material for: Attenuation of Alzheimer’s brain pathology in 5XFAD mice by PTH1-34, a peptide of parathyroid hormone
Source: Alzheimers Res Ther. 2023 Mar 14;15:53. doi: 10.1186/s13195-023-01202-z (PMC10012528; doi:10.1186/s13195-023-01202-z)
Supplement: Supplementary file 2 — Additional file 2: Supplemental file 1. Two-way ANOVA analysis results. [file 13195_2023_1202_MOESM2_ESM.pdf]

Table 1. Two-Way ANOVA analysis of bone phenotypes in Figure 1.

Analysis Item	Source	DF	MS	F	P Value
Tb. BV/TV	$PTH_{1\text{-}34}$ treatment	1	0.05523	437.8	<0.0001
	Genotype	1	0.001296	10.27	0.0125
	Interaction	1	0.00341	27.03	0.0008
Tb. N	$PTH_{1\text{-}34}$ treatment	1	0.6101	24.63	0.0011
	Genotype	1	0.01696	0.6847	0.4320
	Interaction	1	0.05512	2.226	0.1741
Tb. Th	$PTH_{1\text{-}34}$ treatment	1	0.003104	43.89	0.0002
	Genotype	1	4.32e-006	0.06108	0.8110
	Interaction	1	0.0003308	4.677	0.0625
Cb. BV/TV	$PTH_{1\text{-}34}$ treatment	1	0.0009541	5.536	0.0465
	Genotype	1	0.0006308	3.66	0.0921
	Interaction	1	3.333e-005	0.1934	0.6717
Serum Osteocalcin	$PTH_{1\text{-}34}$ treatment	1	441.9	106.4	<0.0001
	Genotype	1	42.26	10.18	0.0128
	Interaction	1	110.4	26.59	0.0009
Serum PYD	$PTH_{1\text{-}34}$ treatment	1	0.03663	48.91	0.0001
	Genotype	1	0.09487	126.7	<0.0001
	Interaction	1	0.01274	17.01	0.0033

Source: Source of Variation, DF: Degrees of Freedom, MS: Mean Square, F: F-statistic, $^*P < 0.05$ significance.

Table 2. Two-Way ANOVA analysis of behavior tests of female mice in Figure 2.

Analysis Item	Source	DF	MS	F	P Value
NOR (Discrimination Index)	$PTH_{1\text{-}34}$ treatment	1	0.02056	1.646	0.2122
	Genotype	1	0.05092	4.078	0.0553
	Interaction	1	0.07966	6.379	0.0189
Y maze (Total Arm Entries)	$PTH_{1\text{-}34}$ treatment	1	7.302	0.1293	0.7225
	Genotype	1	17.44	0.3088	0.5838
	Interaction	1	37.72	0.6678	0.4222
Y maze (Spontaneous Alternation)	$PTH_{1\text{-}34}$ treatment	1	531.5	6.832	0.0155
	Genotype	1	333.2	4.283	0.0499
	Interaction	1	187.4	2.409	0.1343
MWM (Latencies)	Training Time	4	3882	109	<0.0001
	Groups	3	1397	12.92	<0.0001
	Interaction	12	38.89	1.092	0.3765
MWM (Time in Target Quadrant)	$PTH_{1\text{-}34}$ treatment	1	0.00186	9.489e-005	0.9923
	Genotype	1	60.68	3.095	0.0918
	Interaction	1	218.6	11.15	0.0028
MWM (Target Zone Crossovers)	$PTH_{1\text{-}34}$ treatment	1	3.832	1.008	0.3257
	Genotype	1	27.98	7.363	0.0124
	Interaction	1	20.58	5.417	0.0291

Source: Source of Variation, DF: Degrees of Freedom, MS: Mean Square, F: F-statistic, *P < 0.05 significance.

Table 3. Two-Way ANOVA analysis of Aβ deposition in Figure 3.

Analysis Item	Source	DF	MS	F	P Value
Plaque Density (Ctx)	Gender	1	14457	53.21	<0.0001
	PTH_{1-34} treatment	1	15282	56.25	<0.0001
	Interaction	1	3036	11.17	0.0027
Plaque Density (Hipp)	Gender	1	1250	13.44	0.0012
	PTH_{1-34} treatment	1	5092	54.78	<0.0001
	Interaction	1	466.7	5.021	0.0346
Plaque Size (Ctx)	Gender	1	3403	6.524	0.0174
	PTH_{1-34} treatment	1	9742	18.68	0.0002
	Interaction	1	416.5	0.7986	0.3804
Plaque Size (Hipp)	Gender	1	12280	23.46	<0.0001
	PTH_{1-34} treatment	1	20432	39.04	<0.0001
	Interaction	1	8481	16.2	0.0005

Source: Source of Variation, DF: Degrees of Freedom, MS: Mean Square, F: F-statistic, *$P < 0.05$ significance.
