# Supplementary figures and images for: Attenuation of Alzheimer’s brain pathology in 5XFAD mice by PTH1-34, a peptide of parathyroid hormone
Source: Alzheimers Res Ther. 2023 Mar 14;15:53. doi: 10.1186/s13195-023-01202-z (PMC10012528; doi:10.1186/s13195-023-01202-z)

**Fig 4E**

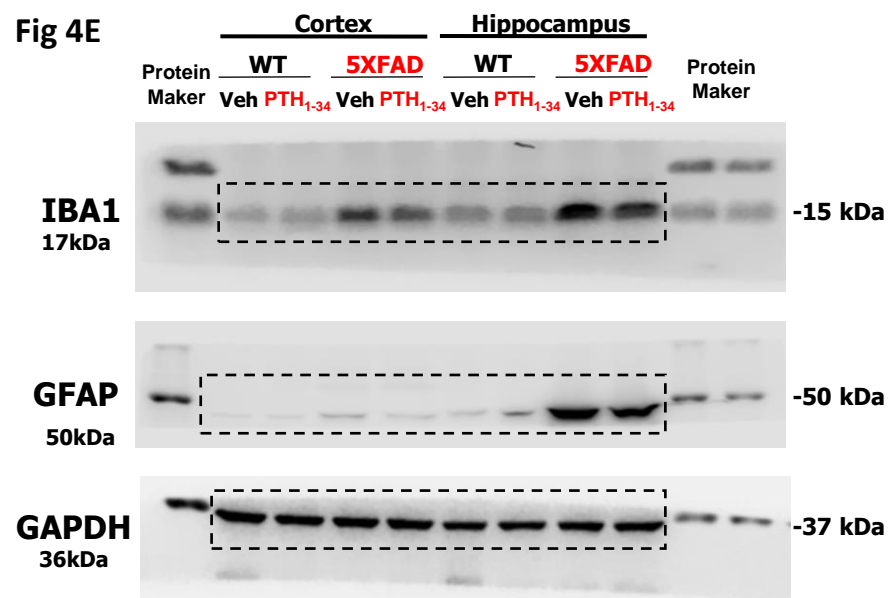

**Fig S6A**

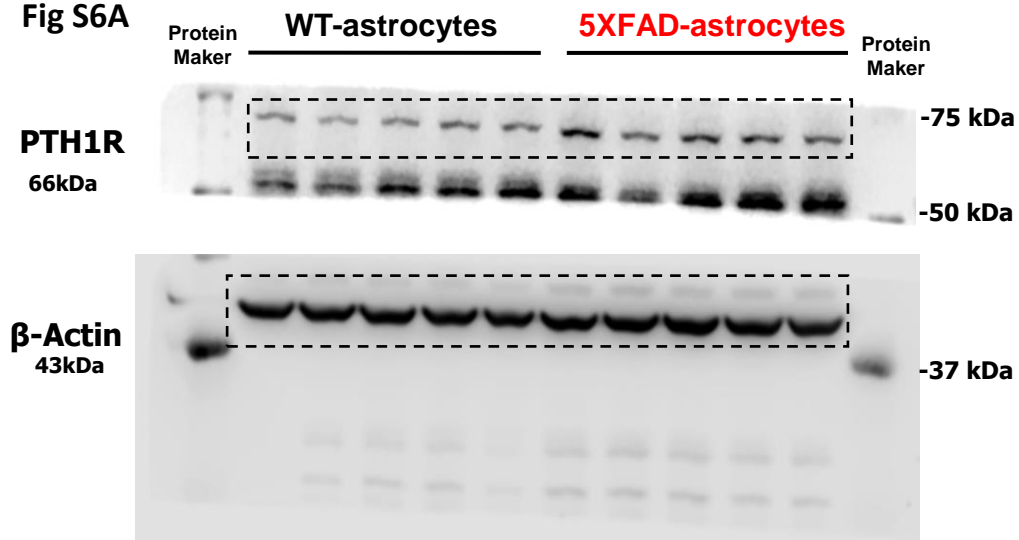

**Fig S7B**

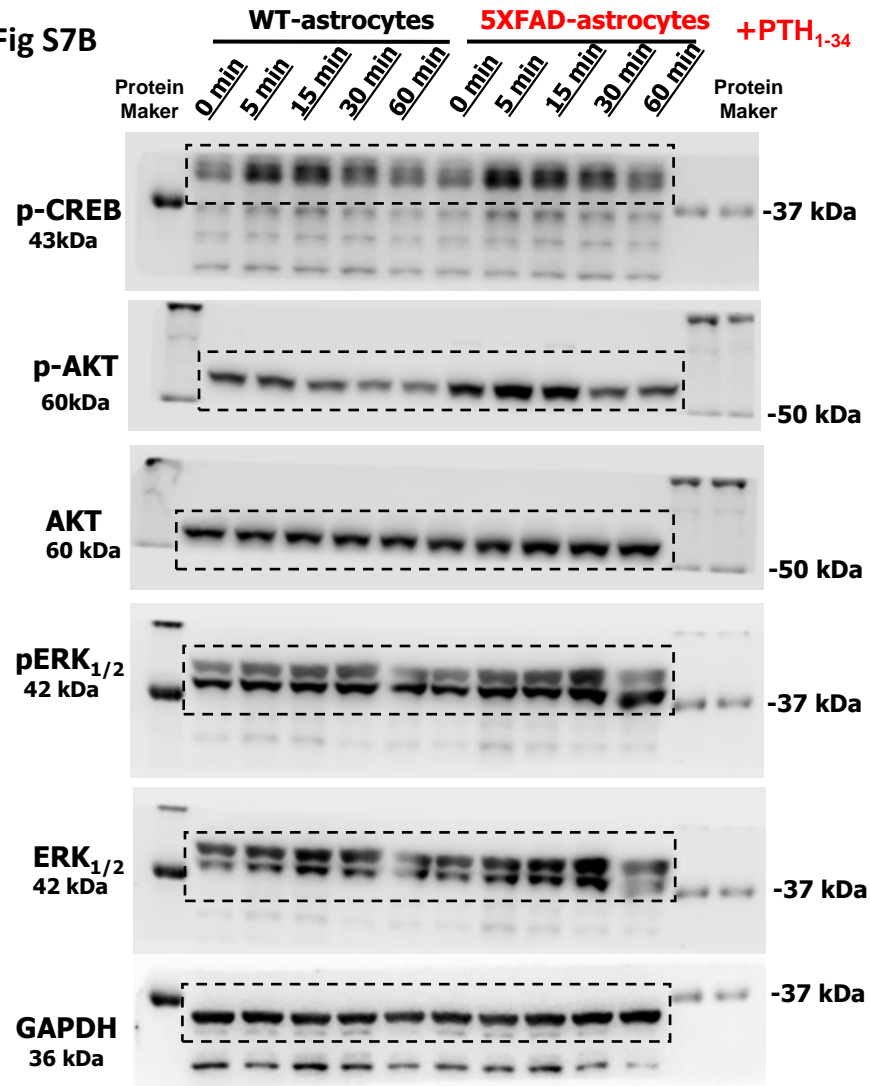

Fig 8E

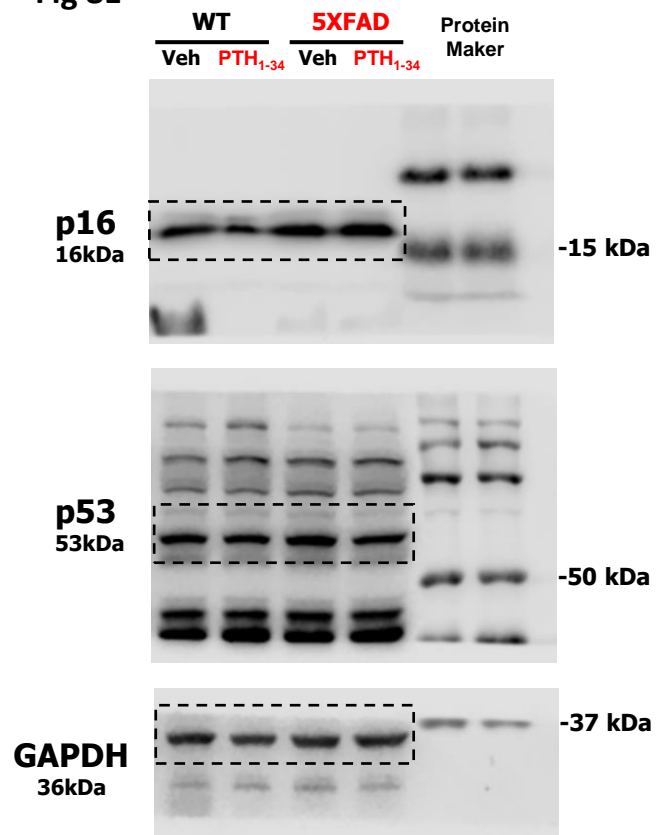

Supplement: Supplementary file 3 — Additional file 3: Supplemental file 2. Full western blots. [file 13195_2023_1202_MOESM3_ESM.pdf]
